# Body Mass Index (BMI) Impacts Soil Chemical and Microbial Response to Human Decomposition

**DOI:** 10.1128/msphere.00325-22

**Published:** 2022-09-22

**Authors:** Allison R. Mason, Hayden S. McKee-Zech, Katharina M. Hoeland, Mary C. Davis, Shawn R. Campagna, Dawnie W. Steadman, Jennifer M. DeBruyn

**Affiliations:** a Department of Microbiology, University of Tennessee, Knoxville, Tennessee, USA; b Department of Anthropology, University of Tennessee, Knoxville, Tennessee, USA; c Department of Chemistry, University of Tennessee, Knoxville, Tennessee, USA; d Department of Biosystems Engineering and Soil Science, University of Tennessee, Knoxville, Tennessee, USA; University of Michigan-Ann Arbor

**Keywords:** human decomposition, soil, microbial ecology, body mass index, carrion ecology, taphonomy, soil microbiology

## Abstract

Microorganisms are key decomposers of vertebrate mortalities, breaking down body tissues and impacting decomposition progress. During human decomposition, both extrinsic environmental factors and intrinsic cadaver-related factors have the potential to impact microbial decomposers either directly or indirectly via altered physical or chemical conditions. While extrinsic factors (e.g., temperature, humidity) explain some variation in microbial response during human decomposition in terrestrial settings, recent work has noted that even under the same environmental conditions, individuals can have different decomposition patterns, highlighting the potential for intrinsic factors to impact microbial decomposers. The goal of this study was to investigate the effects of several intrinsic factors (age, sex, diseases at time of death, and body mass index [BMI]) on chemical and microbial changes in decomposition-impacted soils. In a field study conducted at the University of Tennessee Anthropology Research Facility, soils were collected from the decomposition-impacted area surrounding 19 deceased human individuals through the end of active decomposition. Soil physicochemical parameters were measured, and microbial (bacterial and fungal) communities were assessed via amplicon sequencing. BMI was shown to explain some variation in soil pH and microbial response to human decomposition. Hierarchical linear mixed (HLM) effects models revealed that BMI category significantly explained variation in pH response within decomposition-impacted soils over time (HLM *F* = 9.647; *P* < 0.001). Additionally, the relative abundance of soil *Saccharomycetes* in decomposition soils under underweight donors displayed little to no changes (mean maximum change in relative abundance, +6.6%), while all other BMI categories displayed an increased relative abundance of these organisms over time (normal, +50.6%; overweight, +64.4%; and obese, +64.6%) (HLM *F* = 3.441; *P* = 0.11). Together, these results reveal intrinsic factors influencing decomposition patterns, especially within the soil environment, and suggest BMI is an important factor for controlling decomposition processes.

**IMPORTANCE** This work begins to address questions about interindividual variation in vertebrate decomposition attributed to intrinsic factors, that is, properties of the carcass or cadaver itself. Most research on factors affecting decomposition has focused on the extrinsic environment, such as temperature or humidity. While these extrinsic factors do explain some variation in decomposition patterns, interindividual variability is still observed. Understanding how intrinsic factors influence microbial decomposers will help reveal the ecological impacts of decomposition. This work also has forensic applications, as soil chemical and biological changes have been suggested as indicators of postmortem interval. We reveal factors that explain variation in the decomposition environment that should be considered in these estimates. This is particularly important as we consider the implications of variations in human populations due to diet, age, BMI, disease, toxicological loading, etc. on forensic investigations dealing with decomposing remains.

## INTRODUCTION

Carcass decomposition is an important ecosystem process, stimulating biological activity and nutrient cycling in the local environment. In terrestrial settings, nutrient-rich fluids from decomposing animal carcasses are flushed into the surrounding soil during decomposition, altering soil chemistry and increasing soil microbial activity. Changes in soil chemistry include increased electrical conductivity, ammonium concentrations, and dissolved organic carbon and nitrogen content (1–3). Soil microbes also respond to decomposition products, resulting in altered community composition and activity. This includes decreased alpha diversity, and increased relative abundance of *Firmicutes* and *Bacteroidetes* during human decomposition (4, 5).

While general patterns in soil responses to human decomposition have been identified, variability in the direction and magnitude of soil responses has been noted. For example, change in soil pH is highly variable between studies. While Perrault and Forbes (6) and Aitkenhead-Peterson et al. (7) reported decreased pH in decomposition-impacted soils, other studies have reported increased pH (3, 8–10). It is unknown why differences in soil pH response are observed between sites and studies. However, given that pH is a key abiotic control on microbial communities, this variation in soil chemical response likely impacts the activity and succession of soil microbial decomposers during decomposition (11) and may constrain the fate of carcass-derived carbon and nutrients in the ecosystem.

**FIG 1:**
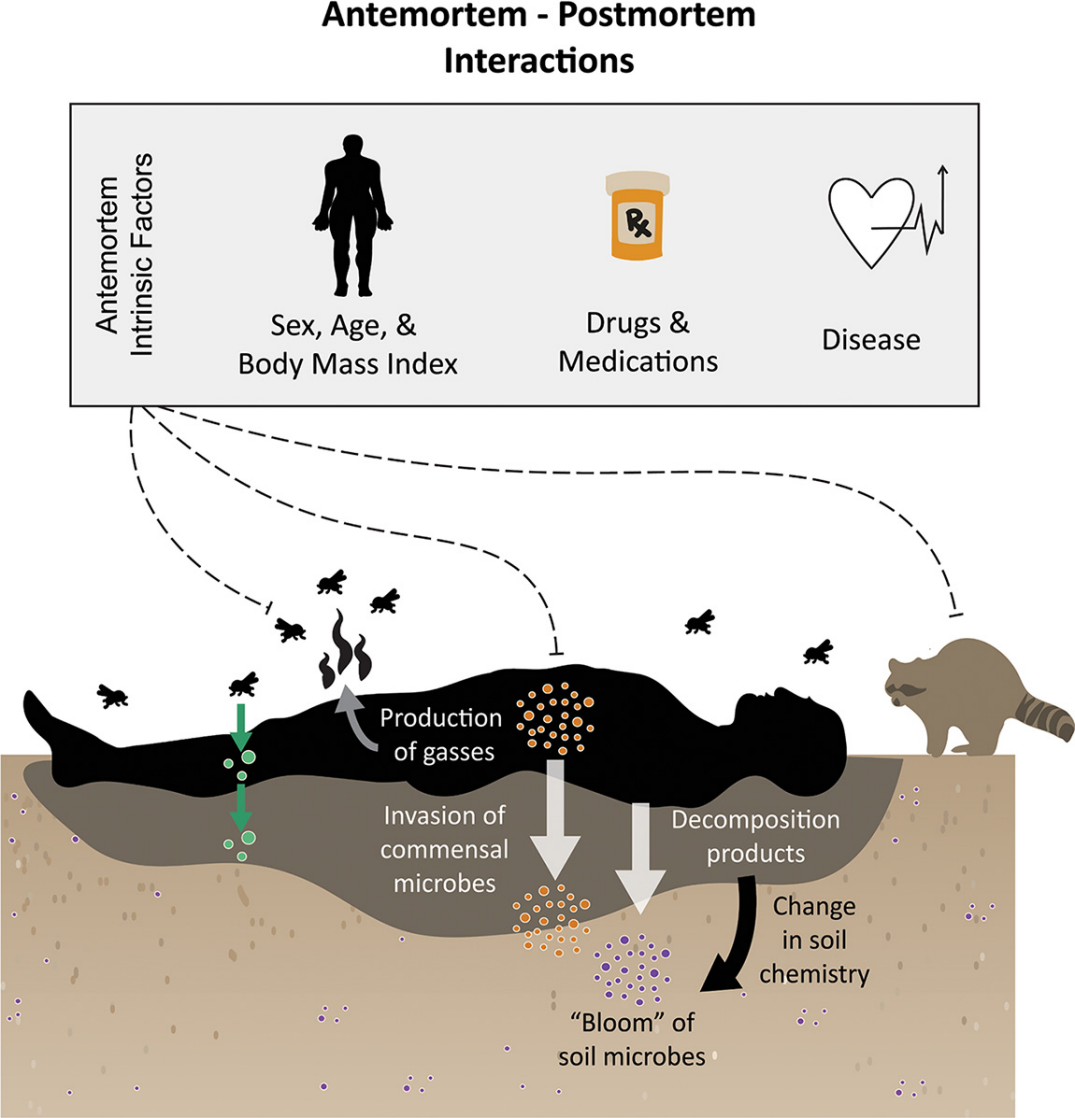
During human decomposition, host-associated microbes, environmental microbes, insects, and scavengers work together to break down body tissues. Liquified decomposition products are flushed into soil where microbes respond to the influx and changes in soil chemistry. Antemortem conditions, such as body mass, age, diet, diseases, or drugs and other treatments, can influence decomposer (i.e., scavengers, insects, and microbes) activity leading to variability in decomposition rate and progression.

Both extrinsic environmental factors (e.g., temperature, season, moisture, etc.) and intrinsic cadaver-related factors (e.g., sex, diseases, mass, etc.) have the potential to impact decomposition rates and patterns. Specifically, warmer temperatures increase insect and microbial activity, and more rapid decomposition rates are observed compared to cooler temperatures (12). Moisture also affects decomposition by mediating microbial activity (13). However, even when extrinsic factors are controlled for, such as when multiple carcasses start decomposition at the same time and experience the same local environmental conditions, differential decomposition patterns have been observed among individuals (1, 2, 14), suggesting that intrinsic factors may also impact decomposition patterns. Genetics, age, sex, diet, body mass index (BMI), and diseases (including therapeutic interventions) can lead to differences in body physiology or chemistry and microbiome between individuals (15–20) that may ultimately impact decomposer activity during decomposition. This could be a direct effect on soil physiochemistry and microbes because of changes in tissue decomposition product chemistry, or an indirect effect, for example, by influencing insect or scavenger behavior, which then alters decomposition progression (Fig. 1).

Changes in soil chemistry and microbial succession in response to human decomposition have been suggested as potential markers to estimate the postmortem interval (PMI) as evidence in death investigations (21–23); therefore, understanding sources of variation in human decomposition is critical for forensic applications. Both extrinsic and intrinsic factors are sources of variability that can impact PMI; however, a majority of decomposition studies have focused only on extrinsic factors (1, 10, 24–27). A few terrestrial decomposition studies have shown that the inclusion of BMI in soil chemistry-based PMI models improves model prediction (2, 28). In contrast, microbial abundance-based PMI models (23, 29) have not incorporated any extrinsic or intrinsic variables in model construction and rely solely on sequencing data. Additionally, the few studies addressing the effects of intrinsic factors on microbial succession concentrate on internal microbial communities (30–32), leaving questions about the influence of intrinsic factors on environmental microbes during decomposition.

The purpose of this study was to investigate the effects of several intrinsic factors (age, sex, diseases at time of death, and BMI) on chemical and microbial changes in decomposition fluids and soils impacted by the fluids during human decomposition. Our overarching hypothesis was that differences in these intrinsic factors would lead to changes in the magnitude of soil chemical response and altered microbial successional patterns. Based on the demographics of our study population (age ≥40), BMI is a reasonable proxy for body composition, where a higher BMI is associated with a greater proportion of fat tissue relative to muscle tissue (33, 34). We hypothesized that differences in the fat:muscle composition of a body changes the nutrient resources available to the environment during decomposition and therefore may alter microbial communities (e.g., a higher fat content might result in selection for lipid metabolizers). Additionally, we examined the effects of diseases on decomposition. Diseases are known to alter microbiome composition and tissue chemistry antemortem (18); thus, we hypothesized that they would also affect the postmortem diversity and activity of microbial decomposers. To address our questions, 19 deceased human individuals were studied in an outdoor surface decomposition experiment at the University of Tennessee Anthropology Research Facility (ARF) from February 2019 to March 2020. Soil chemical analyses were combined with characterization of soil bacterial and fungal communities using amplicon sequencing to link these differences to intrinsic factors, ultimately gaining a better understanding of soil dynamics during human decomposition.

## RESULTS

### BMI and decomposition time.

BMI of the 19 individuals used in this study ranged from 14.2 to 55.1, with a median BMI of 24.6. In general, as BMI of the individual increased, the time (in accumulated degree hours [ADH]) to complete active decomposition increased (linear mixed-effects model *F* = 39.58; *P* < 0.001) (Fig. S1A in the supplemental material). ADH to complete active decomposition ranged from 1,500 for the lowest BMI donor to 18,500 for the highest BMI donor, where the completion of active decomposition was determined as the stage when the abdomen was completely collapsed, and the body was no longer actively producing visible decomposition fluids. While not significant, males in this study had slightly higher BMIs than females (Wilcoxon *W* = 34; *P* = 0.4002), and BMI decreased with age at death (ANOVA *F* = 3.121; *P* = 0.095) (Fig. S1B and C). Differences in BMI by end-of-life diseases were only observed for cardiovascular diseases (*W* = 10, *P* = 0.019), where donors with cardiovascular diseases were associated with higher BMI (Fig. S2).

10.1128/msphere.00325-22.1 FIG S1 Donor body mass index (BMI) impacts total time to complete active decomposition (A). Specifically, total ADH is greater for high BMI individuals. Relationships between donor age (B) and sex (C) and BMI for the study population are shown. In panel B, BMI decreases with age, while in panel C, males (blue) have slightly higher mean BMI compared to females (green), but the difference is not significant. In panels A and B, the red line represents the linear trend between variables and the gray shaded area is the 95% confidence internal. Time is presented in accumulated degree hours (ADH). Download FIG S1, TIF file, 2.4 MB. Copyright © 2022 Mason et al. 2022 Mason et al. https://creativecommons.org/licenses/by/4.0/ This content is distributed under the terms of the Creative Commons Attribution 4.0 International license.

10.1128/msphere.00325-22.2 FIG S2 Comparison of the BMI between donors with (yellow) and without (grey) different illnesses (cancer [A], diabetes [B], cardiovascular [C], respiratory [D], neurological [E], and pneumonia [F]). Red X indicates group means, and *P* values were derived via Wilcoxon nonparametric tests. Download FIG S2, TIF file, 2.5 MB. Copyright © 2022 Mason et al. 2022 Mason et al. https://creativecommons.org/licenses/by/4.0/ This content is distributed under the terms of the Creative Commons Attribution 4.0 International license.

### Decomposition effects on soil physiochemistry and microbial activity.

Decomposition significantly increased soil electrical conductivity (EC) compared to control soils but had no consistent or significant effect on soil pH (hierarchical linear mixed [HLM] effects model pH *P* = 0.149; EC *P* = 0.002) (Fig. S3). When normalized to the control soils (to account for background variability), the log response ratio of soil EC significantly increased over time (*F* = 27.93; *P* < 0.001). In contrast, the log response ratio of soil pH was not significantly different over time (*F* = 0.242; *P* = 0.623) due to high variability between donors. In particular, while a majority of donors (*n* = 14) displayed decreases in soil pH, some donors (*n* = 5) resulted in increased pH (Fig. S3). Similar to EC, decomposition resulted in increased heterotrophic respiration during decomposition (*F* = 11.39; *P* = 0.029). The log response ratio of soil parameters did not significantly differ between seasons or due to the sex of the individual (Table 1). It was also noted that at the end of active decay, all the soil parameters measured were still altered compared to initial conditions.

**TABLE 1:** Analysis of variance results from hierarchical linear mixed-effects models for the log response ratio of each soil parameter and bacterial and fungal community Chao1 richness and inverse Simpson^*a*^

Factors	ADH	BMI category	Sex	Season	ADH: BMI category	ADH: sex	BMI category: sex	ADH: BMI category: sex
pH LRR								
*F*	0.242	***5.392***	0.001	1.880	***9.647***	0.017	***3.833***	***3.066***
*P*	0.623	***0.017***	9.81	0.208	***<0.001***	0.897	***0.045***	***0.029***
Electrical conductivity LRR								
*F*	***27.93***	***2.659***	0.145	0.195	1.709	0.645	0.604	0.247
*P*	***<0.001***	***0.050***	0.704	0.899	0.235	0.439	0.613	0.861
Heterotrophic respiration LRR								
*F*	***11.39***	0.640	0.173	0.550	0.353	0.010	0.211	0.016
*P*	***0.029***	0.595	0.680	0.652	0.796	0.922	0.888	0.904
β-Glucosidase LRR								
*F*	1.117	0.250	0.844	0.583	0.578	3.655	1.923	1.364
*P*	0.296	0.860	0.379	0.643	0.632	0.062	0.179	0.265
*N*-acetyl-β-d-glucosaminidase LRR								
*F*	0.364	0.519	1.487	1.173	***3.096***	***6.157***	2.500	***4.945***
*P*	0.549	0.677	0.249	0.379	***0.035***	***0.016***	0.107	***0.004***
Alkaline phosphatase LRR								
*F*	1.44	0.583	0.005	0.031	0.875	0.188	0.236	0.476
*P*	0.235	0.636	0.945	0.992	0.460	0.670	0.870	0.701
Leucine amino peptidase LRR								
*F*	1.844	1.803	0.179	0.595	2.444	0.056	0.509	0.298
*P*	0.201	0.158	0.674	0.621	0.131	0.817	0.678	0.826
16S Chao1								
*F*	***5.944***	0.1612	0.121	0.108	1.334	0.665	0.041	0.932
*P*	***0.018***	0.920	0.725	0.953	0.274	0.419	0.988	0.432
16S inverse Simpson								
*F*	***5.335***	1.400	0.432	0.015	***3.705***	2.239	0.478	0.009
*P*	***0.025***	0.287	0.525	0.997	***0.017***	0.141	0.703	0.999
ITS Chao1								
*F*	***15.00***	0.081	0.136	0.662	0.899	0.056	0.056	0.524
*P*	***<0.001***	0.970	0.718	0.598	0.448	0.799	0.982	0.667
ITS inverse Simpson								
*F*	***5.700***	1.100	0.005	0.815	0.957	0.006	0.923	1.075
*P*	***0.021***	0.370	0.943	0.522	0.420	0.939	0.447	0.368

a Only samples ≤5.000 ADH were used to capture the linear section of parameter responses. Significant (*P* < 0.05) models are indicated in bold italics. 16S, bacterial; ITS, fungal; LRR, log response ratio.

10.1128/msphere.00325-22.3 FIG S3 Comparison of pH (row 1), electrical conductivity (row 2), heterotrophic respiration (row 3), and extracellular enzyme activities (rows 4 to 7) between control (column 1) and donor (column 2) soils and normalized log response ratio values (column 3) over time. Trends for each donor (names “TOX001 to TOX020”) are delineated by line color. Time, in ADH, has been normalized across donors as percent ADH. Percent ADH for a single sample is defined as the sample ADH divided by total ADH required to complete active decomposition, specific to the donor. Download FIG S3, TIF file, 2.3 MB. Copyright © 2022 Mason et al. 2022 Mason et al. https://creativecommons.org/licenses/by/4.0/ This content is distributed under the terms of the Creative Commons Attribution 4.0 International license.

To assess soil microbial activity in soils, we measured the activity of four common soil extracellular enzymes: β-glucosidase (BG), *N*-acetyl-β-d-glucosaminidase (NAG), phosphatase (PHOS), and leucine aminopeptidase (LAP). Decomposition significantly altered the activity of NAG (*F* = 6.523; *P* = 0.012); however, no significant differences were detected between decomposition and control soils over time for BG (*F* = 0.626; *P* = 0.431), PHOS (*F* = 2.300; *P* = 0.133), and LAP (*F* = 0.054; *P* = 0.817) in decomposition-impacted soils within our data set. Additionally, decomposition time (in ADH) did not significantly describe changes in the log response ratio (LRR) of all four enzymes (Table 1). However, general patterns show NAG and PHOS activity increased (Fig. S3), while the protein-degrading enzyme LAP was variable between individuals (*n* = 13 decreased) in soils during decomposition (Fig. S3).

**FIG 2:**
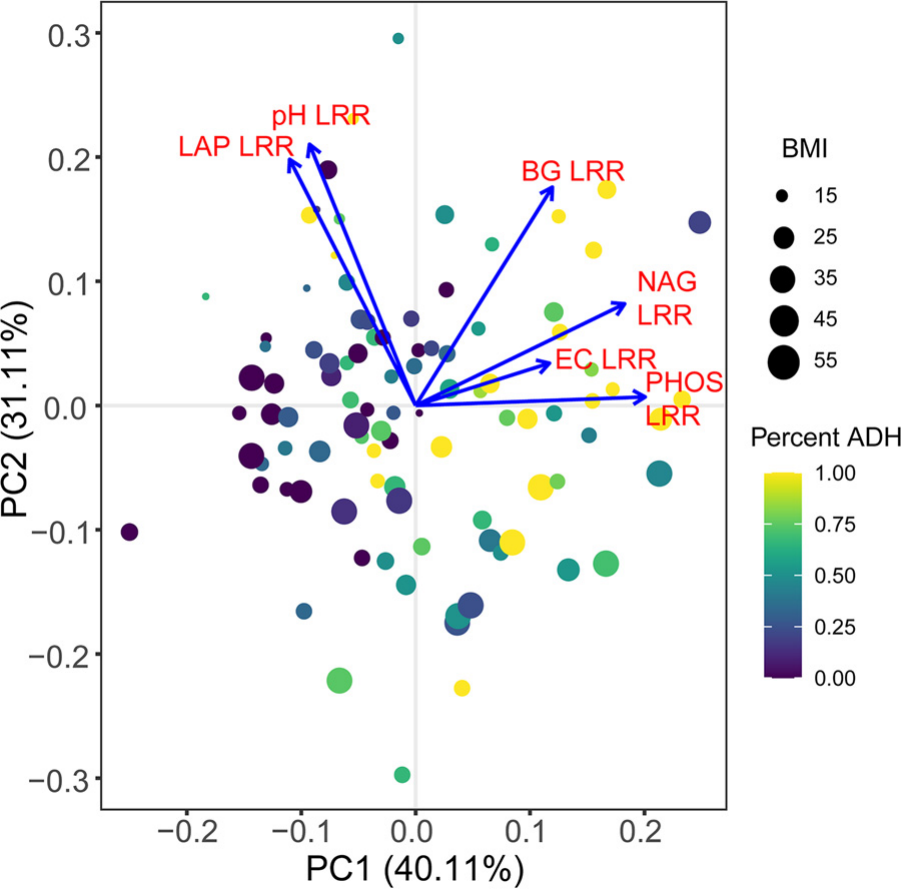
Principal component analysis (PCA) of soil chemical profiles during decomposition. Blue arrows represent principal component (PC) loadings for each variable included in the analysis. Point size corresponds to donor BMI while color denotes sample time as a percentage of the total time required for that donor to complete active decomposition in ADH.

Principal-component analysis (PCA) of normalized (LRR) soil parameters (pH, EC, BG, NAG, PHOS, and LAP) revealed relationships between soil parameter responses during decomposition (Fig. 2). Principal component (PC) 1 was associated with time and related to an increase in soil parameters EC, PHOS, NAG, and BG. Soil response varied along PC2, which was related to differences in pH and LAP activity between individuals.

### BMI and soil responses.

**FIG 3:**
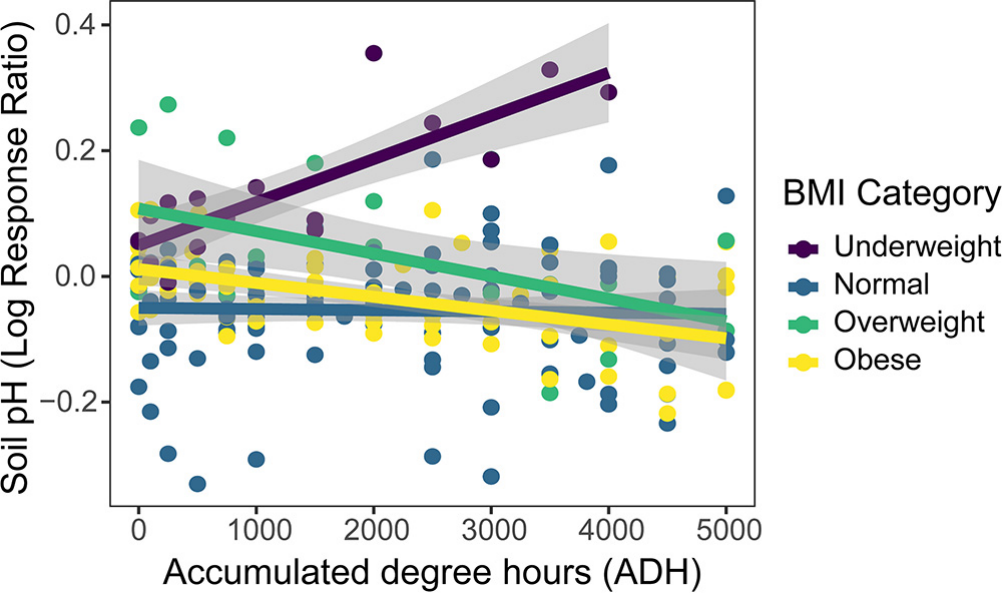
Donor BMI impacts soil pH response during human decomposition. Decomposition of underweight (purple) donors resulted in increased soil pH, while pH decreased during decomposition of normal (blue), overweight (green), and obese (yellow) donors. Lines represent the linear relationship between soil pH and time (in ADH) for each BMI category and gray shading shows the 95% confidence interval for each linear relationship.

We observed a relationship between variability in soil pH response and donor BMI. Hierarchical linear mixed-effects models revealed that BMI category significantly explained variation in pH response within decomposition-impacted soils over time (HLM *F* = 9.647; *P* < 0.001) (Table 1). Specifically, soil pH increased in decomposition-impacted soils of underweight individuals but decreased under normal, overweight, and obese donors (Fig. 3). Soil pH and LAP activity were positively correlated (Spearman *r* = 0.73; *P* < 0.001) during the study period, with LAP activity increasing with pH (*F* = 4.781; *P* = 0.032). However, the change in LAP activity over time (*F = *2.444; *P* = 0.131) was not significantly different between BMI categories.

### Diseases and soil responses.

We sought to determine if donors’ diseases explained some of the variability in soil parameters. The primary effects of the presence and absence of four disease categories (cancer, respiratory diseases, cardiovascular diseases, and neurological diseases) and their influence on soil parameters over time were evaluated with HLM, and results are reported in Table S1. A few trends were observed between donor disease state and soil responses during decomposition. First, pH decreased to a greater extent in the decomposition soil of individuals with neurological diseases compared to those without (HLM *F* = 30.79; *P* < 0.001). Microbial respiration increased to a greater extent for those with cardiovascular diseases over time (*F* = 5.077; *P* = 0.026). Individuals with cancer displayed a greater decrease in soil LAP activity (*F *= 4.201; *P* = 0.045) and less of an increase in microbial respiration (*F* = 8.776; *P* = 0.004) with the progress of decomposition.

10.1128/msphere.00325-22.7 TABLE S1 ANOVA results from linear mixed-effects models testing for the effects of time and the presence/absence of illness categories on respective soil responses. Only samples ≤5,000 ADH were used to capture the linear section of parameter responses. Normalized log response ratio values for each parameter were used in models. *All pH models included BMI as a covariate as it was found to impact pH via HLM. Download Table S1, PDF file, 0.1 MB. Copyright © 2022 Mason et al. 2022 Mason et al. https://creativecommons.org/licenses/by/4.0/ This content is distributed under the terms of the Creative Commons Attribution 4.0 International license.

### Microbial communities.

16S rRNA gene (V4 region) sequencing yielded a total of 17,372,327 raw reads. Removal of primers, erroneous reads (≥1 ambiguous bases, 50 > bp > 275), sequences that aligned poorly, and chimera and nonbacterial sequences resulted in 10,844,074 remaining reads. In R, control samples (e.g., extraction blanks) and singletons were removed leaving 10,579,899 reads across 134 samples with a mean library size of 78,954 reads. Reads were clustered into 33,363 operational taxonomic units (OTUs) at 97% similarity, with a mean of 3,958 OTUs per sample. Good’s coverage for bacterial libraries was greater than 0.994 for all samples; therefore, libraries were randomly subsampled to the smallest 16S library size (*n* = 26,780) before alpha- and beta-diversity analyses.

Gene amplification of the ITS2 region yielded a total of 13,044,056 raw reads. Removal of primers, erroneous reads (≥1 ambiguous bases, bp < 200), and chimera and nonfungal sequences resulted in 9,052,099 remaining reads. After the removal of control samples and singletons in R, 9,037,408 reads across 134 samples remained, with a mean library size of 67,443. Reads were clustered into 13,536 operational taxonomic units (OTUs) at 97% similarity, with a mean of 852 OTUs per sample. For all fungal libraries, Good’s coverage was greater than 0.991. Libraries were randomly subsampled to the smallest ITS library size (*n* = 13,193) before alpha- and beta-diversity analyses.

### Decomposition fluid microbial communities.

Chao1 richness estimates of the decomposition fluid bacterial and fungal communities ranged from 228.93 to 739.95 and 53.49 to 381.38, respectively, while inverse Simpson diversity values ranged from 4.31 to 24.87 and 1.08 to 47.07, respectively. Generally, alpha diversity (Chao1 richness and inverse Simpson diversity estimate) of decomposition fluid bacterial communities increased with greater total ADH to complete active decomposition. Mean richness was 318.3 for donors that completed decomposition quickly (ADH ≤5,000) and 517.1 for donors that took longer to decompose (ADH ≥10,000). Other than total ADH, no variables tested explained the variation in Chao1 or inverse Simpson estimates of decomposition fluid bacterial communities between donors, while none of the variables tested significantly explained fluid fungal community diversity changes (Table S2). Furthermore, no extrinsic or intrinsic factors tested, such as season, age, sex, BMI, and respective diseases, significantly explained (permutational multivariate analysis of variance [PERMANOVA] *P* > 0.05) the variation in bacterial or fungal community composition of decomposition fluid communities (Table S2).

10.1128/msphere.00325-22.8 TABLE S2 ANOVA results from linear models testing for the individual effects various intrinsic and extrinsic factors on bacterial and fungal Chao1 richness and inverse Simpson estimates of decomposition fluid samples (top) and PERMANOVA results from Bray-Curtis distances for bacterial and fungal decomposition fluid communities. PERMANOVA were only assessed for categorical variables, and as a result numeric continuous variables were not assessed (reported as NA). Download Table S2, PDF file, 0.1 MB. Copyright © 2022 Mason et al. 2022 Mason et al. https://creativecommons.org/licenses/by/4.0/ This content is distributed under the terms of the Creative Commons Attribution 4.0 International license.

**FIG 4:**
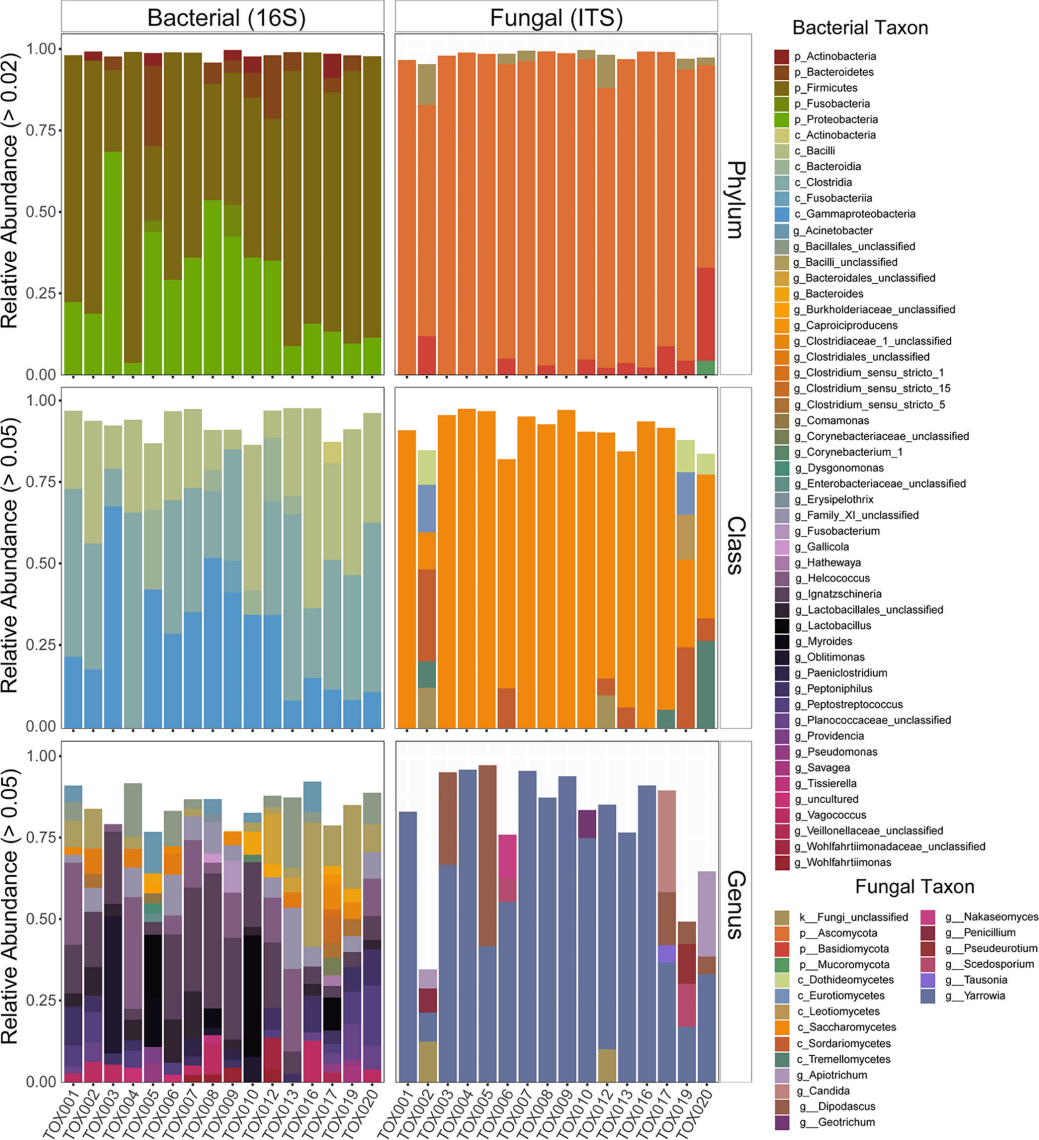
Relative abundance of bacterial (column 1) and fungal (column 2) taxa in decomposition fluid samples at the phylum (row 1), class (row 2), and genus (row 3) levels.

Microbial communities found in the decomposition fluid emitting from the donors were comprised of two to five different bacterial phyla (Fig. 4). A majority of the bacterial communities were comprised of *Firmicutes* and *Proteobacteria* with a mean relative abundance across individuals of 63% and 28%, respectively. At the class level, decomposition fluid communities were dominated by *Clostridia* (39%), *Gammaproteobacteria* (29%), and *Bacilli* (28%). High interindividual variability was observed between fluid bacterial communities at the genus level; however, *Oblitimonas* (25%), *Lactobacillus* (22%), and *Ignatzchineria* (20%) were commonly observed. Fungal communities in the decomposition fluid were comprised of one to four dominant phyla (Fig. 4) depending on the individual. Across all individuals, the fungal phylum *Ascomycota* dominated the decomposition fluid community, with an average relative abundance of 91% across individuals. A majority of these *Ascomycota* belonged to the fungal class *Saccharomycetes* (78%) (Fig. 4); however, a subset of individuals (*n* = 7) also contained *Sordariomycetes* in their decomposition fluids. Aside from one individual (TOX002), a majority of fungal organisms in decomposition fluid (mean 65%) were of the genus *Yarrowia*, a *Saccharomycete*. The genus *Dipodascus* was also present in fluid samples from a subset of individuals (*n* = 5). Interestingly, the common human fungal commensal *Candida* (class *Saccharomycetes*) was only detected in the decomposition fluid of one individual (TOX017) at relative abundances higher than 5%.

### Soil microbial communities.

Soil bacterial communities had greater diversity than decomposition fluid communities (6 times higher Chao1 and 6.5 times higher inverse Simpson index). In general, species richness and diversity of soil bacterial and fungal communities decreased as decomposition progressed (Fig. S4). The contribution of intrinsic and extrinsic factors to interindividual variability in diversity was investigated using HLM. Factors of interest included season, body mass index (BMI), sex, and the presence/absence of different diseases at time of death (Table 1 and Table S1). We found that neither season nor sex had a significant effect (*P* > 0.05, Table 1) on Chao1 richness or inverse Simpson estimates. BMI category was not related to bacterial richness; however, there were differences in diversity (inverse Simpson estimates) over time by BMI category (HLM *F* = 3.705; *P* = 0.017). Specifically, diversity in soils below underweight and normal individuals decreased, while diversity in soils below overweight and obese individuals remained constant through 5,000 ADH. BMI category did not explain differences in soil fungal richness or alpha diversity (Table 1).

10.1128/msphere.00325-22.4 FIG S4 Chao 1 (column 1), inverse Simpson (column 2), and richness (column 3) estimates for bacterial (row 1) and fungal (row 2) communities in soil samples over time (ADH). Boxplot color denotes sample time point. Download FIG S4, TIF file, 1.9 MB. Copyright © 2022 Mason et al. 2022 Mason et al. https://creativecommons.org/licenses/by/4.0/ This content is distributed under the terms of the Creative Commons Attribution 4.0 International license.

Changes in soil chemistry were related to some of the differences in microbial communities over time. Within bacterial communities, richness (*F* = 13.09; *P* < 0.001) and diversity (*F* = 6.331; *P* = 0.014) estimates decreased as soil EC increased. For fungal communities, richness (*F* = 5.424; *P* = 0.023) and diversity (*F* = 17.19; *P* < 0.001) increased with increasing LAP activity in decomposition soils. Fungal community diversity was also shown to increase with increasing BG activity (*F* = 12.41; *P* < 0.001) and soil moisture (*F* = 30.06; *P* < 0.001).

Some trends were observed with microbial richness (Chao1 estimates) and disease (Table S1). Specifically, change in bacterial Chao1 estimates over time differed between donors with and without respiratory (*F* = 4.640; *P* = 0.035) or neurological (*F* = 5.237; *P* = 0.026) diseases. While no overall relationship between Chao1 estimates and the presence of cancer was observed using HLMs, we did observe lower richness in decomposition soils surrounding individuals with cancer compared to those without cancer at one time point: 4,500 ADH (Wilcoxon *P* = 0.01). The presence or absence of disease at time of death did not explain differences in fungal richness or diversity (Table S1). We did not observe any relationship between the presence of cancer, cardiovascular, respiratory, or neurological diseases and bacterial diversity in decomposition soils.

### Soil microbial community structure.

**FIG 5:**
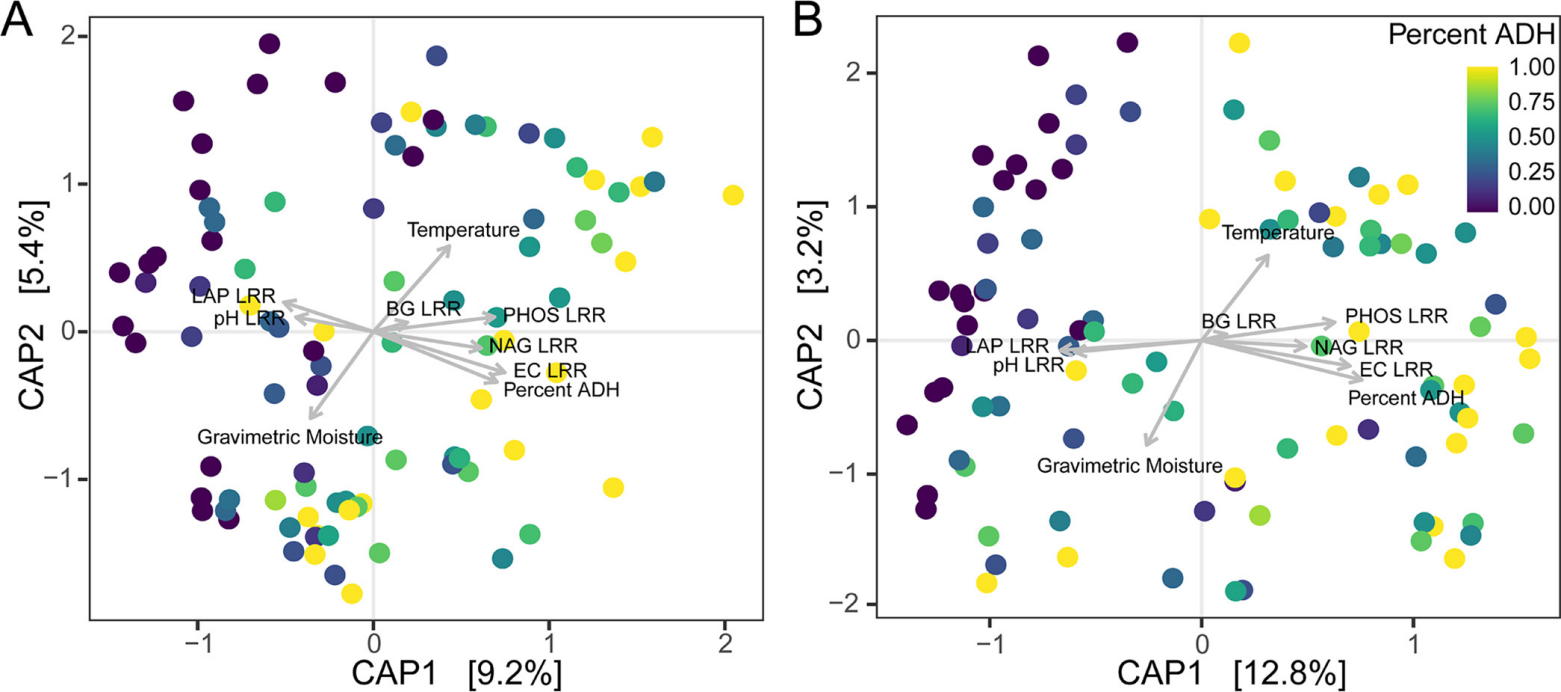
Constrained analysis of principal coordinates (CAP) of Bray-Curtis distances in bacterial (A) and fungal (B) community structure within decomposition soil samples. Gray arrows represent the influence of each environmental parameter included in the analysis. Data point color denotes decomposition time as percent ADH (sample ADH divided by total ADH required to complete active decomposition).

Microbial community composition was altered in response to human decomposition (Fig. 5A and B). Both bacterial (PERMANOVA *F* = 2.25; *r*^2^ = 0.025; *P* = 0.004) and fungal (*F* = 2.97; *r*^2^ = 0.023; *P* = 0.002) community composition was significantly different in decomposition-impacted soils compared to controls. Constrained analysis of principal coordinates (CAP) of Bray-Curtis distances was used to relate changing microbial community composition to soil environmental parameters. The first two CAP axes explained 14.6% and 16% of bacterial and fungal community variation, respectively (Fig. 5A and B). In both bacterial (Fig. 5A) and fungal (Fig. 5B) CAP analyses, gravimetric moisture and temperature were related to CAP2, while pH, EC, extracellular enzymes, and time (as percent ADH) were related to CAP1. Interestingly, the relationships between soil chemical variables and microbial compositional shifts followed those identified in the soil chemical responses during decomposition (Fig. 2). Microbial community composition shifted along CAP1, which was strongly correlated with increased EC in decomposition soils. Similar to soil chemistry shifts, soil pH and LAP activity were positively correlated in constrained ordinations. Composition also varied along CAP2, where soil samples with increased temperature had lower gravimetric moisture.

**FIG 6:**
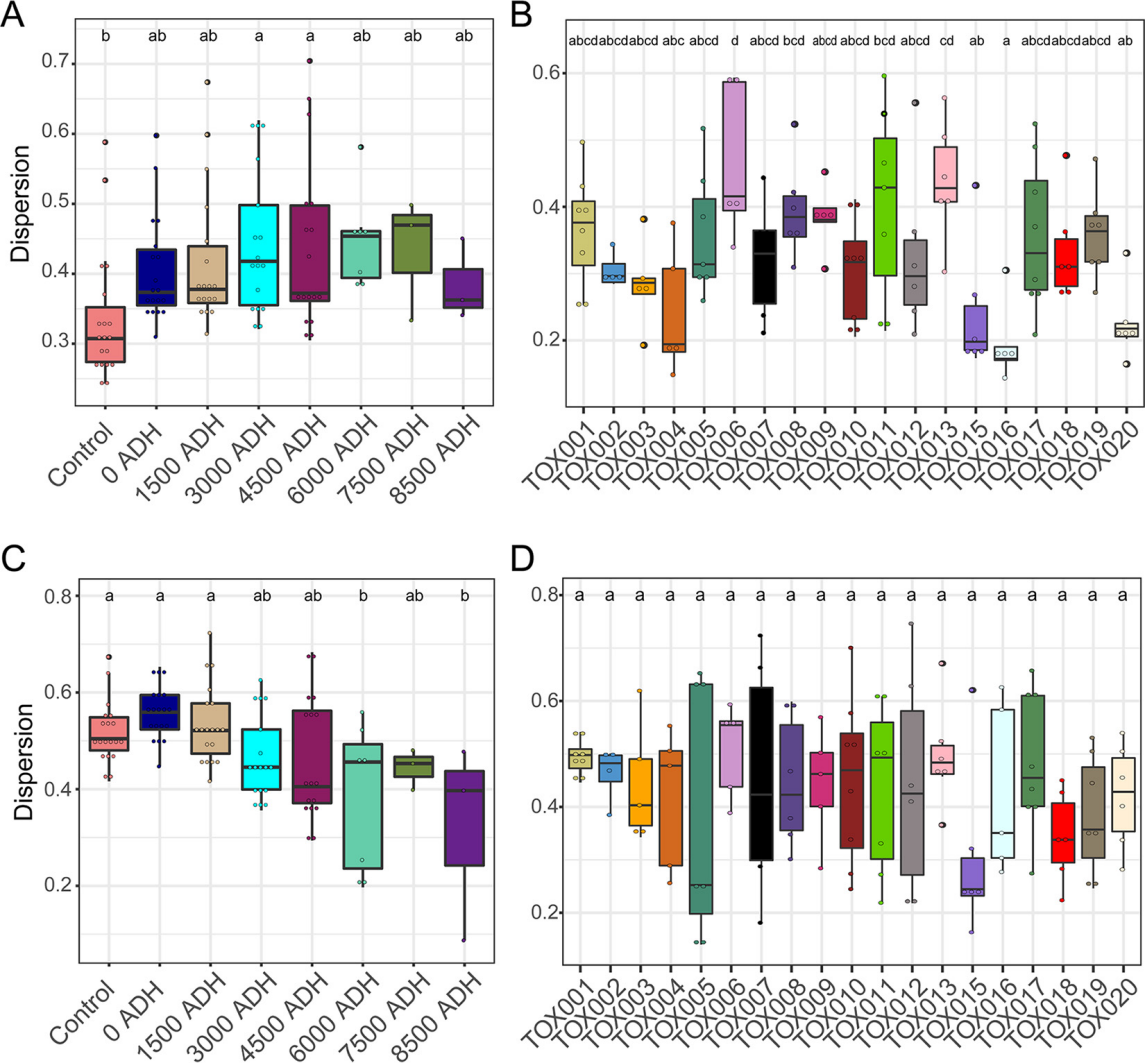
Bacterial (A, B) and fungal (C, D) community dispersion differs during active decomposition. Beta dispersion increases with time in bacterial communities (A) but decreases in soil fungal communities (C). Additionally, dispersion between donors differs for bacterial communities (B), while remaining similar across donors for fungal communities (D). In panels A and C, color denotes sample time point, while in B and D color represents donor. Letters above boxplots are the result of *post hoc* Tukey tests where differences denote significant differences between groups (*P* < 0.05). ADH, accumulated degree hours.

Some differences in bacterial and fungal compositional shifts in response to decomposition were observed. Specifically, while soil bacterial communities changed as decomposition progressed, they did not become more similar with time. Statistical beta dispersion, a measure of variability between groups, shows that the statistical beta dispersion of bacterial communities increases slightly over time (Fig. 6A). In contrast, dispersion of fungal communities decreased with time (beta dispersion *F* = 11.17; *P* = 0.001) (Fig. 6C), showing that fungal communities become more similar during active decomposition. Additionally, interindividual variability was greater within bacterial communities than within fungal communities (Fig. 6B and D). Beta dispersion of bacterial communities was significantly different between individuals (*F* = 4.239; *P* = 0.001), while the beta dispersion of fungal communities was not (*F* = 0.796; *P* = 0.711).

Two-way PERMANOVAs assessing the effects of time and respective factors on bacterial and fungal community composition were performed, while beta-dispersion results for all variables are reported in Table S3. Within bacterial communities, all variables were significant (PERMANOVA *P* < 0.05), while all but cancer were significant for fungal communities. This was likely due to repeated measures within each donor causing lower *P* values. As a result, we also evaluated potential effects on community composition within respective time points (0, 1,500, 3,000, 4,500, and 6,000 ADH) using one-way PERMANOVA with each factor. An individual’s age explained some of the variation in bacterial (PERMANOVA *F* = 1.611; *P* = 0.017) and fungal (*F* = 1.318; *P* = 0.0489) communities at 1,500 ADH (Table S4). No other variables were significant for bacterial communities, but season did significantly impact soil fungal communities. Specifically, variation in fungal community composition in decomposition-impacted soils was partially explained by season at 3,000 and 4,500 ADH. Additionally, fungal communities differed by season (*F* = 1.271; *P* = 0.033) in control soils, while bacterial communities did not (*F* = 1.124; *P* = 0.2707). We also noted that soils collected from decomposition plots before placement were not significantly different from controls in regard to bacterial (*F* = 1.217; *P* = 0.2328) and fungal (*F* = 1.148; *P* = 0.1958) community composition.

10.1128/msphere.00325-22.9 TABLE S3 Beta-dispersion results for various intrinsic and extrinsic factors for bacterial and fungal communities. Download Table S3, PDF file, 0.1 MB. Copyright © 2022 Mason et al. 2022 Mason et al. https://creativecommons.org/licenses/by/4.0/ This content is distributed under the terms of the Creative Commons Attribution 4.0 International license.

10.1128/msphere.00325-22.10 TABLE S4 PERMANOVA results assessing the effects of and various respective intrinsic and extrinsic factors on soil bacterial and fungal community composition within the selected timepoints (0, 1,500, 3,000, 4,500, and 6,000 ADH). Preplacement samples included controls and 0 ADH samples and were compared to see if control soils differed from decomposition sites before donor placement. Download Table S4, PDF file, 0.1 MB. Copyright © 2022 Mason et al. 2022 Mason et al. https://creativecommons.org/licenses/by/4.0/ This content is distributed under the terms of the Creative Commons Attribution 4.0 International license.

### Soil bacterial community composition.

**FIG 7:**
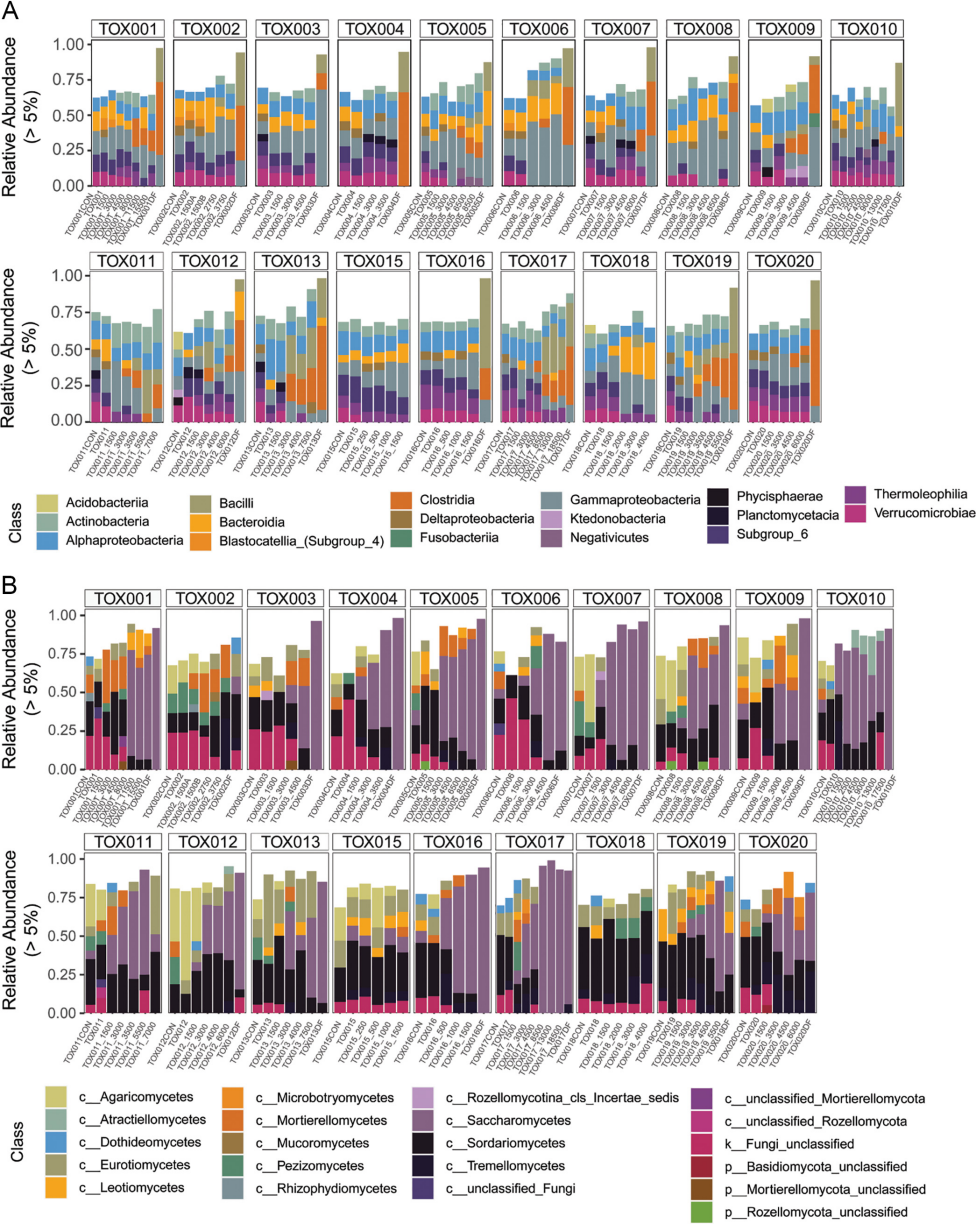
Relative abundance of bacterial (A) and fungal (B) classes over time (*x* axis) for each of the 19 individuals in the study, labeled TOX001 to TOX020.

Relative abundance of bacterial taxa in soil changed throughout decomposition (Fig. S5A and Fig. 7A). At the phylum level, increases in relative abundance of *Proteobacteria* (median 13.8% increase across all donors) and *Firmicutes* (20.4%) were observed over time, while median relative abundance of *Verrucomicrobia* (median 7.7% decrease across all donors) and *Acidobacteria* (12%) decreased. Changes in *Bacteroidetes* and *Actinobacteria* were variable among individuals: *Bacteroidetes* increased in 14 individuals and decreased in five, while *Actinobacteria* increased in 11 and decreased in 8. At the class level, *Gammaproteobacteria* (median 15.7% increase across all donors), *Clostridia* (9.5%), *Actinobacteria* (7.2%), and *Bacilli* (6.9%) increased in decomposition soils (Fig. 7A); notably, these were taxa also detected in fluid samples (Fig. 4). Relative abundance of taxa in class *Verrucomicrobiae* (median 7.7% decrease across all donors) and *Thermoleophilia* (6.1%) decreased in decomposition-impacted soils, while *Bacteroidia* response was variable (*n* = 13 individuals increased, *n* = 5 decreased, and *n* = 1 no change). At the genus level, the soil organism Subgroup_6_ge within the phylum *Acidobacteria* was found before cadaver placement and decreased (median 6.6% decrease across all donors) during active decomposition, while Acinetobacter increased (median 8.6% increase across all donors).

10.1128/msphere.00325-22.5 FIG S5 Relative abundance of bacterial (A) and fungal (B) phyla over time (x axis) for each of the 19 individuals in the study, labeled TOX001 to TOX020. Download FIG S5, TIF file, 2.2 MB. Copyright © 2022 Mason et al. 2022 Mason et al. https://creativecommons.org/licenses/by/4.0/ This content is distributed under the terms of the Creative Commons Attribution 4.0 International license.

### Soil fungal community composition.

Fungal community structure in decomposition-impacted soils also changed over time. Prior to placement, soil fungal communities included the phyla *Ascomycota*, *Basidiomycota*, *Mortierellaomycota*, and *Rozellomycota*, among others. As decomposition progressed, members of *Ascomycota* (median 29% increase across all donors) and *Mortierellomycota* (+4%) increased in relative abundance, while members of *Basidiomycota* (median 11% decrease across all donors), *Rozellomycota* (−1%), and *Chytridiomycota* (−1%) decreased (Fig. S5B). At the class level, the most notable change in fungal community structure was an increase in *Saccharomycetes* between 3,000 and 4,500 ADH (median 57% increase across donors) (Fig. 7B), corresponding with the timing of fluid introduction to soil. Within *Saccharomycetes*, we observed increases in the genera *Yarrowia* (median 22% increase across all donors) and *Dipodascus* (23%) associated with the same time period.

### *Saccharomycetes* and BMI.

**FIG 8:**
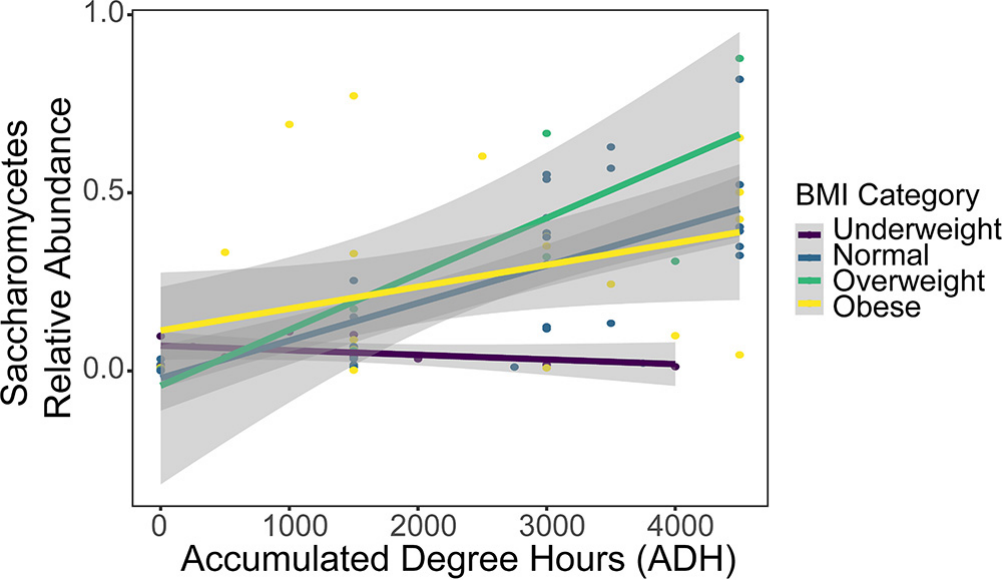
Relative abundance of the fungal class *Saccharomycetes* over time (ADH) in decomposition-impacted soils. Relative *Saccharomycetes* abundance increased as decomposition progresses in soils below normal (blue), overweight (green), and obese (yellow) donors but does not change in soils below underweight (purple) donors. Lines represent the linear relationship between relative *Saccharomycetes* abundance and time (in ADH) for each BMI category (line color), while gray shading is the 95% confidence interval for each linear relationship.

Increases in *Saccharomycetes*, previously observed during outdoor juvenile pig decomposition (35), in decomposition-impacted soils were notably related to donor BMI. In particular, decomposition soils below underweight donors displayed little to no changes in relative *Saccharomycetes* abundance over time (Fig. 8) (HLM *F* = 3.441; *P* = 0.11). This included individuals 002, 015, and 018, whose BMI ranged from 14.2 to 20.2 and had visibly little fat tissue. We also found that as donor BMI increased, changes in pH over time differed (HLM *F* = 8.799; *P* = 0.009) and the maximum relative abundance of *Saccharomycetes* observed in decomposition soils during active decomposition increased (ANOVA *F* = 5.582; *P* = 0.03). None of the other factors tested, including disease categories, season, sex, or age, were found to be related to relative *Saccharomycetes* abundance in decomposition soils (Table S1). Variation in relative *Saccharomycetes* abundance in decomposition soils was also positively related to heterotrophic respiration (HLM *F* = 3.972; *P* = 0.052), but not to any other soil parameter, including pH (*P* > 0.05).

## DISCUSSION

The main goal of this study was to assess the impact(s) of intrinsic, or cadaver-related, factors on soil chemical and microbial patterns during human decomposition. Specifically, we evaluated the relationships between cadaver sex, age, body mass index (BMI), and diseases to soil chemical responses and microbial community structure and activity in decomposition-impacted soils. Data were collected from 19 individuals, and we were able to identify intrinsic factors, including BMI, related to variable chemical responses and microbial activity in decomposition-impacted soil.

Our study observed interindividual variation in soil chemical and microbial responses. Some of this variation was explained by donor BMI, the relationship between an individual’s weight and height. BMI is often used as an indirect measure of body fat in humans, since BMI and percent body fat are typically positively correlated (36). However, BMI is an indirect measure and does not account for variability in body composition (37). For example, an individual with high lean muscle mass and low body fat may have a high BMI. While we did not measure body fat percent in our study, we visually observed that the individuals with high BMI had a greater amount of fat tissue as opposed to lean muscle tissue, so we can assume that BMI generally reflects body fat content for our study sample. Additionally, BMI was calculated from cadaver measurements of height and weight as opposed to values reported in medical history or on a driver’s license. While cadaver height may be problematic due to inaccuracy when positioning the cadaver for measurement, reported antemortem values may not be up to date (38). However, results by Ferorelli et al. (38) suggest differences between antemortem and cadaver height are minimal.

We found that soil pH response varied between individuals, with soil pH increasing for individuals with BMI <18.5 and decreasing for those with BMI >18.5. Variable pH response is in accordance with previous vertebrate decomposition studies in terrestrial ecosystems, where some studies report increased soil pH (1, 3, 9), while others report decreased pH (6, 8, 10). Fancher et al. (2) observed both increased and decreased soil pH within the same study assessing human decomposition. Further, we observed that the direction of soil pH change (increase versus decrease) was partially explained by differences in BMI, suggesting that BMI influences pH response during human decomposition. While BMI has not been linked to any specific soil response during human decomposition previously, Fancher et al. (2) and Aitkenhead-Peterson et al. (28) noted that the inclusion of BMI in models improved the estimation of PMI from multiple soil responses, including pH. Soil pH response may have varied due to relative ratios of fat and muscle decomposition products associated with individuals of different BMIs. Fat tissue contains 60% to 85% lipids (39), which are hydrolyzed into stearic, oleic, and palmitic acid, while decomposition of muscle tissue releases ammonium (NH_4_^+^). Therefore, a greater proportion of acidic products would be expected in individuals with more fat tissue, resulting in decreased soil pH. Alternatively, muscle tissue contains more nitrogen than fat tissue (40), and ammonification of proteins, peptides, and amino acids present in muscle tissue release NH_4_^+^. In individuals with less fat tissue and more muscle, a greater relative proportion of NH_4_^+^ is expected, resulting in increased pH.

pH is arguably one of the most important environmental factors impacting soil microbial communities and activities directly and indirectly (11). In this study, we observed a positive relationship between soil pH and leucine aminopeptidase (LAP) activity during decomposition, suggesting that pH regulates LAP response during human decomposition and/or LAP activity generates additional NH_4_^+^ thereby increasing soil pH. LAP, a general aminopeptidase, has been shown to exhibit optimum activity under alkaline conditions (pH 6 to 8) (41), potentially explaining greater LAP activity in soils of individuals whose pH increased during decomposition. Microbes produce LAP for nitrogen acquisition (42), increasing nitrogen availability for the community. As a result, variable LAP activity may impact the rate of microbial-mediated peptide degradation and nutrient availability for soil microbes, leading to increased variability in microbial community composition and function between individuals. Our results are consistent with previous decomposition studies that recorded soil LAP activity (3, 43). For example, Keenan et al. (3) reported increased pH and LAP activity during terrestrial beaver decomposition, while Keenan et al. (43) observed decreased pH and LAP activity in a multi-individual human grave. DeBruyn et al. (1) conducted a surface decomposition experiment comparing pigs and humans and observed that pH and LAP activity decreased in human decomposition-impacted soil but increased under pigs. Thus, the pH-LAP relationship appears robust, and further work will be needed to determine the mechanism behind this relationship and its implications for the fate of decomposition products.

We also observed that the relative abundance of *Saccharomycetes* in decomposition soils was related to donor BMI. While *Saccharomycetes* relative abundance increased in decomposition soils below normal, overweight, and obese donors, underweight individuals displayed no such trend. Taylor (44) also identified the fungal class *Saccharomycetes* as one of the main taxa responsible for fungal community compositional shifts in decomposition soils below donors ranging in BMI from 24.6 to 29.6 (BMI categories: normal and overweight). *Yarrowia*, a *Saccharomycete*, has also been shown to increase in soils during juvenile pig decomposition (35). The relationship between *Saccharomycetes* relative abundance and BMI may be due to differences in proportions of fat and muscle tissue between individuals. The dominant *Saccharomycetes* genera we observed (*Yarrowia* and *Dipodascus*) are found in a variety of environments, including insects and humans, and display diverse metabolic capabilities (45). Specifically, the *Yarrowia* species are known for their ability to metabolize lipids (45), suggesting that high BMI individuals with more fat provide a favorable substrate, giving *Yarrowia* a competitive advantage over other fungi.

As BMI was related to soil pH changes and pH can impact microbial communities, we thought that the variable soil *Saccharomycetes* response may be related to pH response during decomposition. However, we did not observe a linear relationship between soil pH and *Saccharomycetes* relative abundance in our data set, suggesting the response of *Saccharomycetes* was not due to an altered pH. While many members of *Saccharomycetes*, including Saccharomyces cerevisiae, prefer low pH, *Yarrowia lipolitica* has been suggested to adapt to a wide range of pH conditions (45, 46). As *Yarrowia* and *Dipodascus* were the dominant *Saccharomycetes* genera present in our communities, this adaptability to variable pH conditions may explain why soil pH was not significantly related to *Saccharomycetes* relative abundances in decomposition soils. Interestingly, we noted that relative abundances of *Acidobacteria* also did not appear to have a relationship to the pH changes observed, despite the fact that this group is often reported as increasing relative abundances in low pH soils (47). This could be due their oligotrophic/K-selected growth habits or sensitively to other abiotic or biotic changes occurring in decomposition soils.

The observed relationships between BMI and microbial community composition may be partially explained using ecological stoichiometry theory, which posits that microbial community composition and activity is driven by the balance of the organism’s nutritional requirements and nutrient content of available resources (i.e., C:N) (48, 49). Within this framework, individual taxon responses to disturbance may be predictable when the difference between organismal C:N:P requirement and C:N:P of resources is considered. Release of nutrient-rich fluids from the cadaver into the surrounding soil alters carbon and nitrogen pools during decomposition (9, 50, 51). Yet, C:N of various body tissues differs. Specifically, muscle tissue contains more nitrogen and thus a lower C:N than fat. For example, Keenen et al. (40, 50) recorded a C:N of 49 and 3.3 for fat and muscle tissue, respectively, in beavers (*Castor canadensis*). This difference in C:N between tissue types may have important implications when considering the nutrient composition of different individuals. For example, we can consider two individuals with the same weight and muscle mass, but different body fat percentages (15% versus 30%). Assuming similar elemental composition of fat and muscle across vertebrates, we can use values from Keenan et al. (40, 50) to calculate that the C:N ratio of the fat and muscle fraction of these individuals would be 4.07 and 4.83, respectively (Fig. S6). For a 93-kg individual, this roughly equates to 4.62 kg more carbon for a 30% body fat individual, compared to the 15% body fat individual. While these are clearly rough estimates based on several assumptions, they illustrate that differences in body composition (fat, muscle, and bone) between individuals may alter resource pools and proportions of breakdown products, both of which may impact microbial decomposer presence and activity.

10.1128/msphere.00325-22.6 FIG S6 Calculation of C:N of fat and muscle between individuals with different body fat percentage, here 15% (blue) and 30% (red). Assumptions are outlined in the top gray box, while equations are presented in dashed boxes. Download FIG S6, TIF file, 2.6 MB. Copyright © 2022 Mason et al. 2022 Mason et al. https://creativecommons.org/licenses/by/4.0/ This content is distributed under the terms of the Creative Commons Attribution 4.0 International license.

In addition to a relationship with BMI, we also identified some potential relationships between reported diseases contributing to the individual’s cause of death and soil responses. Donors with cancer at time of death had altered microbial decomposer communities with lower diversity and respiration rates compared to donors without cancer. This could be due to differences in body tissues due to disease or the presence of chemotherapeutics and other drugs (e.g., morphine) in the system. It could also be an indirect effect of an altered microbiome due to the disease and/or therapeutic interventions (e.g., radiotherapy). For example, cancer has been associated with altered gut microbiome composition (18), with some cancer patients having a greater proportion of *Firmicutes* relative to *Bacteroides* (52). Wang et al. (52) observed lowered diversity and decreased *Firmicutes:Bacteroides* ratio after radiotherapy, suggesting cancer treatments and therapeutics alter gut communities further. Thus, cancer may lead to different microbiome composition at time of death, altering decomposer communities and impacting decomposition patterns. Notably, we did not observe differences in bacterial or fungal community composition of fluids released from the body during decomposition between disease categories. This could be because we pooled the collected decomposition fluids throughout decomposition to make a composite sample for each individual, which may have masked any initial variability in microbiomes between individuals. Additionally, because we used a donated population, we had low replication in some of the disease categories, which lowered the statistical power we had to detect potential effects. A further complicating factor is that drugs and therapeutics used were likely not identical between donors with the same diseases. To see if decomposer communities do indeed differ due to disease, increased sample sizes, toxicological screens of donors, characterization of the initial microbiome at time of death, noting length of time to initial fluid release, and time-series analysis of fluid communities would be informative.

One interesting observation we noted was the variation in soil bacterial and fungal community structures during human decomposition. During active decomposition soil fungal community composition shifted, becoming more similar in composition between individuals. In contrast, soil bacterial community composition did not become more similar, suggesting that the soil bacterial community response is more variable than soil fungal response during active decomposition. Additionally, we generally observed greater interindividual variability in bacterial communities compared to fungal communities and seasonal differences in bacterial communities that were not seen in fungal communities. These differences may result from distinct responses to environmental stressors caused by the release of decomposition products due to overall diversity differences between bacterial and fungal groups. This study and others have shown that the soil chemical environment is heavily impacted by vertebrate decomposition (2, 3, 7, 50, 53). In our study, soil EC (correlating to salinity) increased up to 48 times higher than background conditions. This level of disturbance impacts soil microbial communities, altering their structure and activity. Both bacterial and fungal communities make up soil microbial communities; however, differences in their diversity can impact their resistance (i.e., ability to withstand change) and/or resilience (i.e., rate of community recovery) to disturbance events (54). In this study, bacterial diversity was much higher than fungal diversity in our predecomposition soils. As higher diversity can be associated with greater resistance to disturbance events (54), this may explain why bacterial communities did not become more similar during active decomposition. In contrast, fungal communities, with lower diversity, were less resistant to disturbance, resulting in the filtering of those species in unfavorable conditions.

### Limitations/future directions.

There are several aspects of this study that limit our interpretation and provide avenues for future research. First, while our results show a relationship between BMI and soil pH response, it is important to note that BMI is an indirect measure based solely on an individual’s height and weight and does not account for differences in fat proportion or distribution due to sex and age (37). This may explain some of the variability we observed in moderate BMI individuals. A more direct measure of body composition (percentages of fat, muscle, and bone), such as dual-energy X-ray absorptiometry (36), would be needed to evaluate relationships between elemental stoichiometry and microbial responses in future decomposition studies. Second, while we were able to investigate relationships with some individual intrinsic factors, there are other intrinsic factors (e.g., diet, ancestry, and drugs in the system at time of death) that were not considered that may impact decomposition patterns. Additionally, our observations were likely limited by the demography of our sample population: all donors had a mean age of 71. Finally, while we investigated the general effects of disease states, as they contributed to the cause of death, we were not able to look at interactions between diseases or address seasonal effects due to the size of our data set and distribution of donors within disease categories across seasons. As a result, we reported trends that should be investigated further with a restrained and/or larger study population and followed up with a controlled, targeted study to identify the effects of diseases and their therapeutics on decomposition patterns.

### Conclusions.

The goal of this research was to assess the impact(s) of sex, BMI, and disease status at time of death on the soil decomposition environment and microbiome. Our results suggest an individual’s BMI can influence decomposition patterns, leading to interindividual variability in soil chemical and microbial responses during human decomposition. We also observed that the preliminary effects of diseases on decomposition, notably cancer (and/or its therapeutic interventions), may have a potential inhibitory effect on microbial decomposers. Together these results indicate that intrinsic factors, such as BMI, likely play a role in driving decomposition rates and patterns and should be considered in taphonomy studies. Further, our results may have implications for postmortem interval estimation methods that rely on bacterial community data. In particular, even in our demographically narrow study, the decomposer microbiome (or necrobiome) varied between individuals suggesting successional patterns may not be universal when larger sample sizes and more diverse populations are considered. Intrinsic factors helped explain some of this variability, so the inclusion of intrinsic and environmental data may help improve postmortem interval models.

## MATERIALS AND METHODS

### Study design.

A series of human decomposition studies were conducted at the University of Tennessee Anthropology Research Facility (ARF), located in Knoxville, TN (35° 56' 28” N, 83° 56' 25” W). The ARF is a roughly 2-acre area of temperate mixed deciduous forest dedicated to studying human decomposition (43). The soil type at ARF consists of clay loam and channery clay loam overlaying a bedrock of limestone, shale, and sandstone. These soils are classified as Coghill-Corryton complex (CcE), containing 25 to 65% slopes and described as rocky and well drained (https://websoilsurvey.sc.egov.usda.gov/).

Nineteen deceased human individuals (herein called “donors”) were selected from those donated through the ARF body donation program. Donors were selected independent of age, weight, ancestry, or sex. Donors without open wounds were selected to avoid altering insect behavior and/or microbial activity during decomposition. Medical histories and known prescribed medication lists were obtained for all individuals enrolled in the study. Donors ranged in age from 40 to 91 years and were near evenly distributed by sex (Table 2). All donors identified as White. BMI (kg/m^2^) was calculated using cadaver height (m) and weight (kg) recorded upon arrival at the Forensic Anthropology Center (FAC). From this, BMI of donors ranged from 14.2 to 55.1 and was used to group donors into BMI groups using Centers for Disease Control (CDC) categories (Underweight, <18.5; Normal, 18.5 to 24.9; Overweight, 25 to 29.9; and Obese, ≥30). Donors were also categorized by the presence or absence of six broad disease categories, determined by conditions reported as contributing to cause of death. We recognized that categorizing by disease encompasses altered physiology or microbiome due to disease, as well as medications and other therapeutic interventions used to treat the disease. Disease categories included diabetes, cancer, cardiovascular diseases, respiratory diseases, neurological diseases, and pneumonia (Table 2). In most cases, more than one condition was reported as contributing to cause of death; therefore, some donors were attributed multiple conditions.

**TABLE 2:** Demographics for all donors enrolled in study^*a*^

Donor ID	Sex	Age	BMI	BMI category	Season of Placement	Total ADH	Total days	Reported diseases					
								Diabetes	Cancer	Respiratory diseases	Cardiovascular diseases	Neurological diseases	Pneumonia
TOX001	Female	63	33.4	Obese	Winter	15,500	93	Y	N	N	Y	N	N
TOX002	Female	78	20.2	Normal	Spring	3,750	37	N	Y	N	N	N	N
TOX003	Male	71	19.9	Normal	Spring	4,500	27	N	N	Y	N	N	N
TOX004	Female	84	19.3	Normal	Spring	3,500	15	N	Y	N	N	N	N
TOX005	Female	71	23.2	Normal	Spring	8,500	32	N	N	Y	N	N	Y
TOX006	Male	64	24.9	Normal	Spring	4,500	15	N	Y	N	Y	Y	N
TOX007	Male	89	26.2	Over	Spring	6,000	18	N	N	N	Y	N	N
TOX008	Male	40	24.3	Normal	Summer	6,500	21	N	Y	N	N	N	N
TOX009	Female	72	23.8	Normal	Summer	4,500	13	N	Y	N	N	N	N
TOX010	Male	65	54.1	Obese	Summer	17,500	50	N	N	Y	Y	N	Y
TOX011	Male	81	22	Normal	Summer	7,000	19	N	N	N	N	Y	N
TOX012	Female	77	29.4	Over	Summer	6,000	17	Y	N	N	N	Y	N
TOX013	Male	54	41.6	Obese	Summer	7,500	25	N	N	N	N	N	N
TOX015	Male	78	14.2	Under	Fall	1,500	27	N	N	Y	N	N	N
TOX016	Male	62	37.5	Obese	Fall	1,500	57	N	N	Y	N	N	N
TOX017	Female	62	55.1	Obese	Winter	18,500	191	N	Y	N	Y	N	N
TOX018	Female	85	16	Under	Winter	4,000	74	N	N	N	N	Y	N
TOX019	Female	67	19.6	Normal	Winter	5,500	72	N	N	N	N	N	N
TOX020	Male	91	31.2	Obese	Spring	6,000	48	N	N	Y	N	N	N

a Total ADH is the ADH at the end of active decomposition. Disease presence was based on those reported in medical histories as contributing to cause of death. Y, yes; N, no.

Donors were placed within the facility to decompose as they were received between February 2019 and March 2020 (Table 2). Before placement, all individuals were kept at 4°C for no longer than 1 week following death. Each individual was placed supine on the soil surface without clothing. Individuals were placed on soils that had not been exposed to decomposition for at least 6 months. For every donor site, a control site was identified at least 1 m away from the donor and either upslope or at the same elevation. Hourly temperature was monitored using TinyTag temperature and humidity loggers (Gemini Data Loggers, UK) placed within 0.5 m of the donor and 1 m above the ground. Temperature readings were taken every hour from time of placement until the donor was unenrolled from the study at the end of active decomposition. Active decomposition is the decomposition stage characterized by the release of gasses and fluid from the remains, the cessation of fluid purge, and complete collapse of the abdomen (55), all of which were used as indicators of the end of active decomposition here. Accumulated degree hours (ADH) were calculated by summing the hourly temperature (°C) above a threshold over time. In this study, 0 ADH was defined as time of placement within the ARF, and a threshold temperature of 10°C was used for ADH calculations to keep our results consistent with additional insect data collected from these donors.

### Soil sampling.

Soil samples were collected at approximately 0, 100, 250, 500, 750, and 1,000 and thereafter at 500 accumulated degree hour (ADH) intervals until the end of active decomposition when donors were unenrolled from the study. Sampling ADH values were within 50 ADH for time points <1,000 and within 100 ADH for time points >1,000 ADH. For each soil sample, a sterile 10-ml syringe with the tip end cut off (Norm-Ject Henke-Ject syringe) was used to collect five to eight soil cores of 5 cm depth. At each time point, soils were collected from within the area of visibly saturated soils around decomposing donors (≤15.2 cm or 6 in. from the body) and at the control site. Soils were then homogenized by hand and debris larger than 2 mm was removed (i.e., rocks, roots, insects, etc.). A 20-g subsample was weighed into a 4 oz. Whirl-Pak bag (Nasco), flash frozen in liquid nitrogen, and stored at −80°C before DNA extraction. The remaining soil was stored at 4°C before soil physiochemical analyses.

### Decomposition fluid sampling.

Decomposition fluids emitted from the donors were collected as they pooled on the surface of the soil surrounding the donor. Fluid was collected using a sterile 30-ml syringe (BD 30-ml Luer-Lok tip syringe). The tip of the syringe was carefully inserted into the surface of pooling fluid and slowly drawn up. This process was repeated until ~10 ml of fluid was collected. The syringe was then carefully inverted two to three times to homogenize, and fluid was evenly dispensed into four 2-ml cryovials. Cryovials were flash frozen in liquid nitrogen and stored at −80°C before DNA extraction.

### Soil physiochemical analyses.

Soil physiochemical analyses were conducted within 4 days of collection using soils stored at 4°C. Soils were acclimated to room temperature for at least 30 min before all measurements. Soil electrical conductivity (EC) and pH were measured on a 1:2 soil to deionized water mixture by weight using an Orion Star A329 pH/ISE/Conductivity/Dissolved Oxygen portable multiparameter meter (ThermoFisher). Soil gravimetric moisture was calculated after determining the water weight of soil by oven drying duplicate 2- to 3-g soil aliquots at 105°C for 72 h.

### Soil biological activity.

After soil samples acclimated to room temperature, respiration rates were measured via the accumulation of CO_2_ over 24 h as described previously (43). Briefly, 6 g of soil was sealed in 60-ml serum bottles and CO_2_ was measured with duplicate injections, immediately after capping and after 24 h, into a LI-820 CO_2_ analyzer (LI-COR) with manual injection. Incubations were conducted in the dark at room temperature (~20°C).

Measurement of extracellular enzyme activities and 16S rRNA gene amplicon sequencing were performed for all donors on a subset of soil samples. For most donors, 10 soil samples corresponding to the control and decomposition samples at 0, 1,500, 3,000, and 4,500 ADH plus the final (T_f_) sample were used for enzyme assays and DNA extraction, as these ADH values were the median 25, 50, and 75 percentiles across all donors. Some donors completed active decomposition at/or before 4,500 ADH or extended longer (i.e., TOX010 T_f_ = 17,500 ADH), therefore fewer or additional time points were assessed.

The activity of four common soil extracellular enzymes were evaluated: β-glucosidase (BG; sugar degradation), phosphatase (PHOS; phosphorous mineralization), *N*-acetyl-β-glucosaminidase (NAG; chitin degradation), and leucine amino peptidase (LAP; protein degradation). Enzyme assays were conducted in triplicate for all donors as previously described in Bell et al. (56), with some modifications. Briefly, 2.75-g subsamples were weighed from soils stored at −80°C and held at −20°C before assays. Soil samples were then thawed at room temperature and slurried in 50 mM Tris buffer at pH 6.7 (average pH of our soil samples) in a blender (Waring commercial blender, model WF2212114). Assays were conducted using 800 μl of slurry and 200 μl of enzyme substrate (1,500 μM). Optimum substrate concentrations were determined before conducting assays on soil samples and were found to be 1,500 μM for all enzymes. Additionally, standard curves were conducted using MUB and MUC concentrations ranging from 0 μM to 200 μM. All standard curves and soil samples were assessed in triplicate and included blanks for each sample to evaluate background concentrations. All metadata, including soil chemistry, sample information, and corresponding donor information and code for analysis, can be found (https://github.com/jdebruyn/TOX-microbiology).

### DNA sequencing.

DNA was extracted from all soil and decomposition fluid samples using the DNeasy Powerlyzer PowerSoil kit (Qiagen Inc.). Extractions were conducted according to manufacturer’s instructions with the following modifications. Briefly, 0.25 g of soil/fluid was used for all extractions and homogenized (MO BIO PowerLyzer Bench Top Bead-Based Homogenizer) using settings suggested for high organic soils (2,500 RPM for 45 s). DNA was eluted in 100 μl of 10 mM Tris buffer and stored at −20°C prior to sequencing. Total DNA concentrations were determined using the Quant-iT PicoGreen dsDNA assay kit (Invitrogen) using an assay volume of 200 μL and 1 μL of DNA. Each donor’s control soil extracts were pooled, ensuring equal amounts of DNA were added, with the rationale that the pooled extract would capture natural variability over the course of the study. Similarly, each donor’s decomposition fluid extracts were pooled. DNA extracts were sent to the University of Tennessee Knoxville Sequencing Core Facility (Knoxville, TN) for library preparation and sequencing. Both bacterial and fungal communities were sequenced; the primer set 515F (57)/806R (58) was used to amplify the V4 region of the 16S rRNA gene, the ITS2 region in fungi was amplified using primers described previously (59). Libraries were prepared using the Nextera XL DNA library preparation kit (Illumina) and sequenced on the Illumina MiSeq platform to generate paired-end reads. Raw sequences have been deposited to NCBI’s Sequence Read Archive (SRA) under BioProject PRJNA817528.

Reads were processed using Mothur (60) (v.1.43.0). Briefly, low-quality sequences (16S: Q > 20, bp ≤ 50; ITS Q > 20, bp < 200), sequences containing ambiguous bases (≥1), and nonbiological (primers and adapters) sequences were removed. 16S reads were aligned to the SILVA nonredundant database (v132), while ITS was not aligned. VSEARCH was used to remove chimera sequences. Bacterial and fungal sequences were classified using the SILVA nonredundant database (61) (v132) and UNITE RefS database (62) (version 02.02.2020), respectively. Bacterial sequences were then clustered into operational taxonomic units (OTUs) based on ≥97% sequence similarity and the default opticlust method, while fungal sequences were clustered using abundance-based greedy clustering.

### Statistics.

To account for natural variation in soil parameters over time and space and allow for comparison between donors, we normalized measured soil parameter values by calculating the log response ratio (LRR = ln[treatment value/control value]) (63). LRR values greater than 0 were higher in decomposition-impacted soils compared to control soils, while values less than 0 were lower in decomposition-impacted soils compared to control soils.

The effects of time (as ADH), season, BMI, sex, and diseases on the soil parameter’s response during decomposition were assessed with hierarchical linear mixed-effects models, allowing for random slopes and/or intercepts by donor. Random effects terms were chosen for each response variable based on best fit (determined by Akaike information criterion [AIC]). Due to small sample sizes, the effects of diabetes (*n* = 2) and pneumonia (*n* = 2) were not evaluated. Statistical differences were then assessed using a type III analysis of variance (ANOVA) with Satterthwaite’s method. All models were run in R using the *lmer()* function (R package lme4 version 1.1.25) and statistically analyzed using the *anova()* function from lmerTest (version 3.1.3) package. Due to differences in ADH to complete active decomposition, patterns of soil chemical responses differed between donors with some exhibiting linear patterns and others displaying saturating trends. Thus, only data points ≤5,000 ADH were used in hierarchical linear mixed-effects models to capture the linear response period (436 samples across all donors).

Alpha (Chao1 richness and inverse Simpson) and beta (Bray-Curtis dissimilarity) diversity of soil and fluid communities, respectively, were calculated in R and beta diversity was visualized using principal coordinate analysis (PCoA) within the R package phyloseq (version 1.32.0). Changes in alpha diversity within soil communities were assessed using hierarchical linear mixed-effects models and statistical differences assessed using type III analysis of variance (ANOVA) with Satterthwaite’s method. Differences in soil community structure were evaluated with permutational analysis of variance (PERMANOVA), via the *adonis()* function in the R package vegan (version 2.5.6). The functions *betadispr()* and *permutest()*, both from vegan (version 2.5.6), were used to evaluate multivariate homogeneity of dispersions between specified groups (i.e., Donor, ADH) from Bray-Curtis dissimilarity values.

### Data availability.

Raw sequences have been deposited to NCBI's Sequence Read Archive (SRA) under BioProject PRJNA817528. All R code used for this research is available: https://github.com/jdebruyn/TOX-microbiology.
