# Supplementary figures and images for: Body Mass Index (BMI) Impacts Soil Chemical and Microbial Response to Human Decomposition
Source: mSphere. 2022 Sep 22;7(5):e00325-22. doi: 10.1128/msphere.00325-22 (PMC9599287; doi:10.1128/msphere.00325-22)

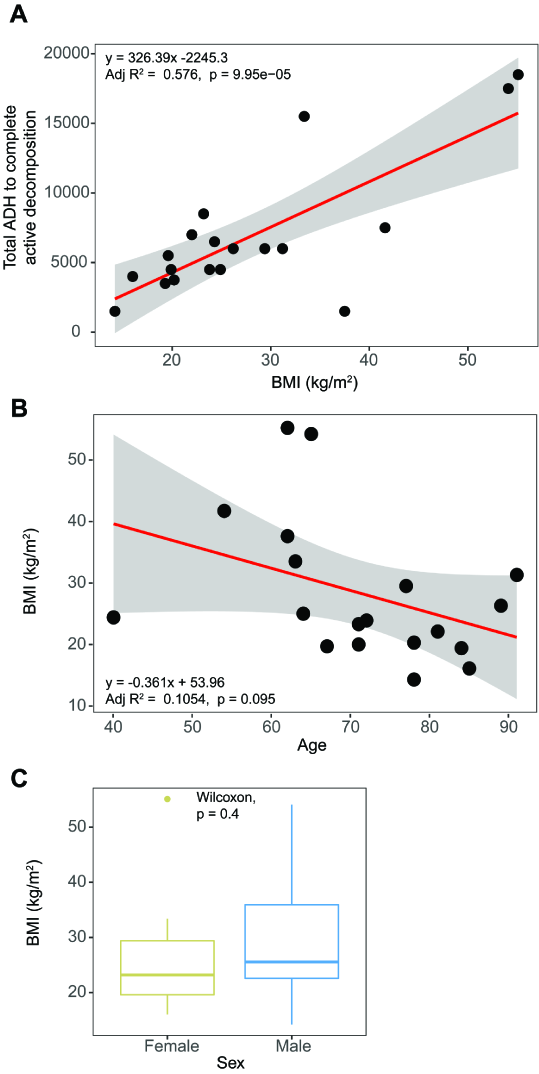

Supplement: FIG S1 [file msphere.00325-22-s0001.tif]

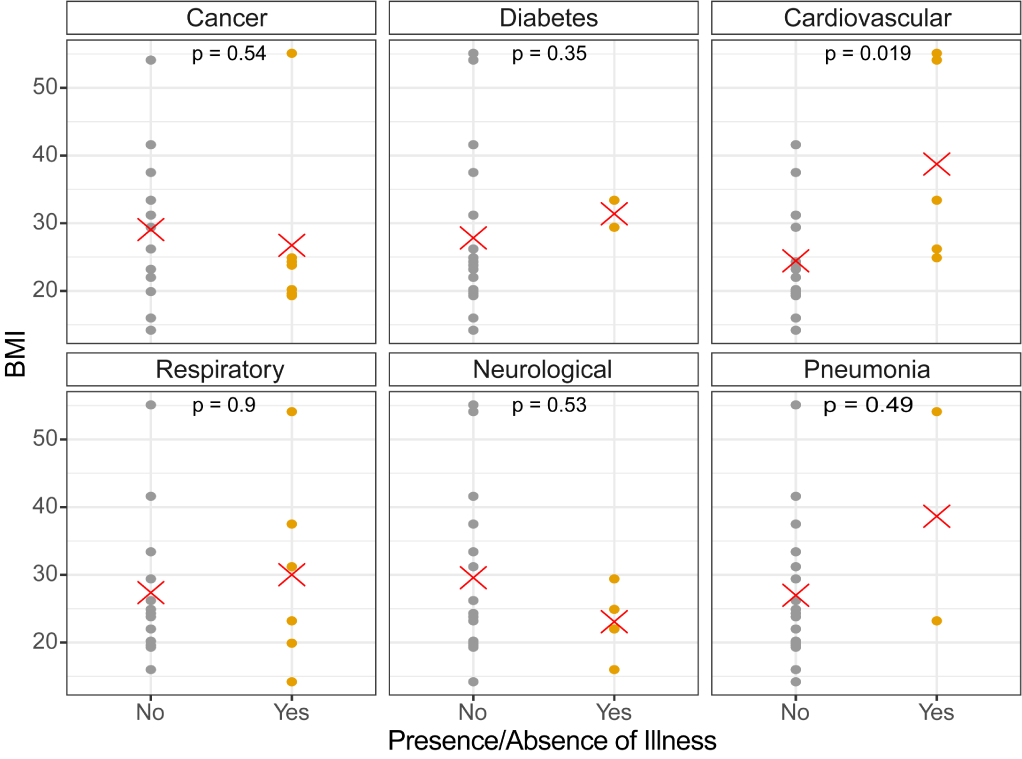

Supplement: FIG S2 [file msphere.00325-22-s0002.tif]

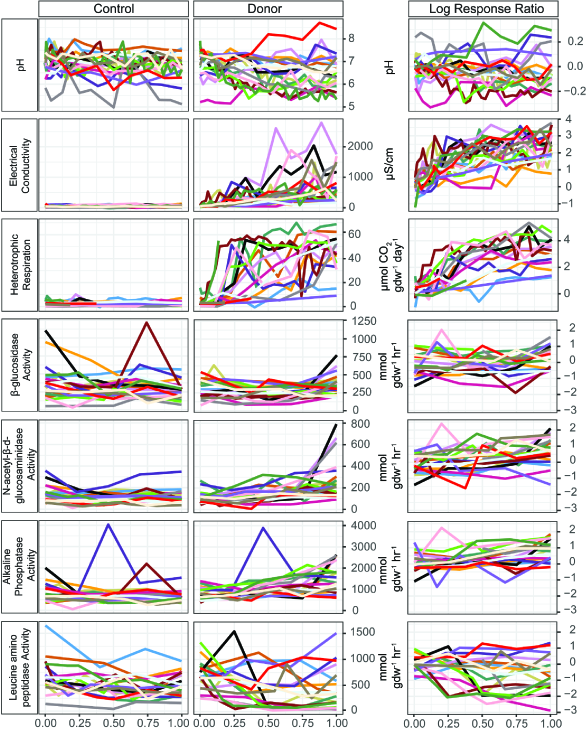

Supplement: FIG S3 [file msphere.00325-22-s0003.tif]

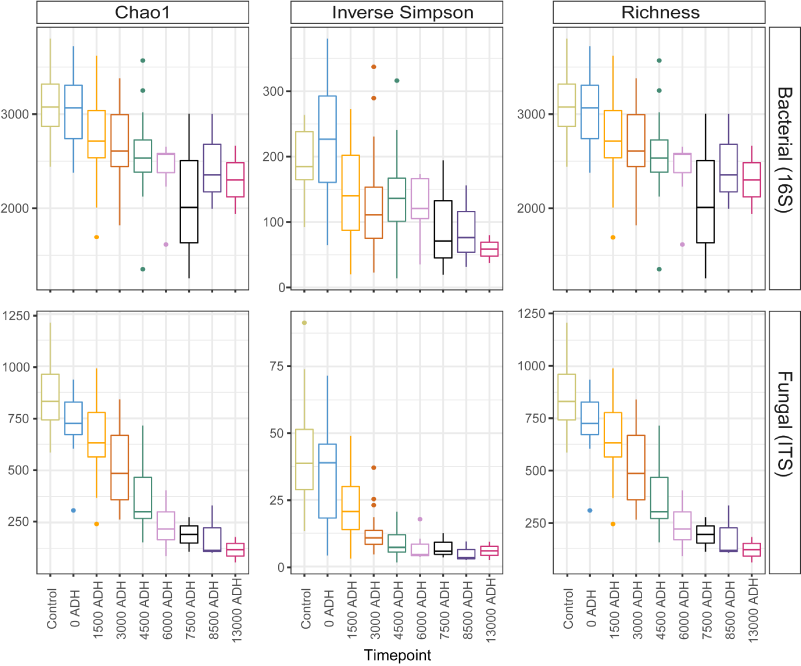

Supplement: FIG S4 [file msphere.00325-22-s0004.tif]

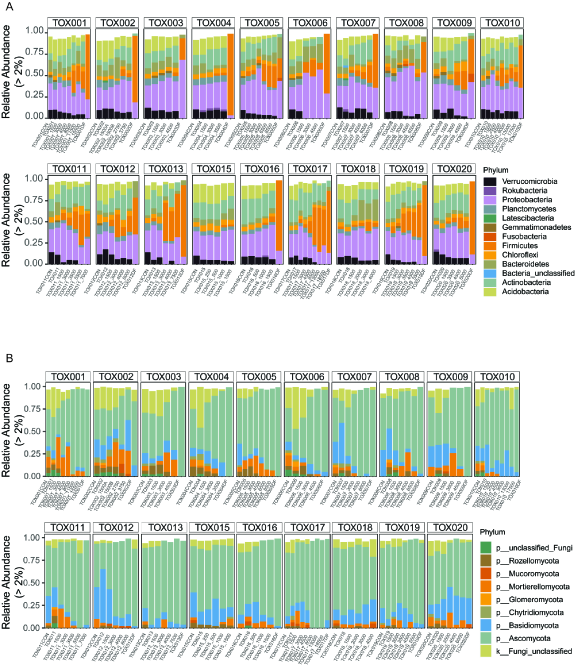

Supplement: FIG S5 [file msphere.00325-22-s0005.tif]

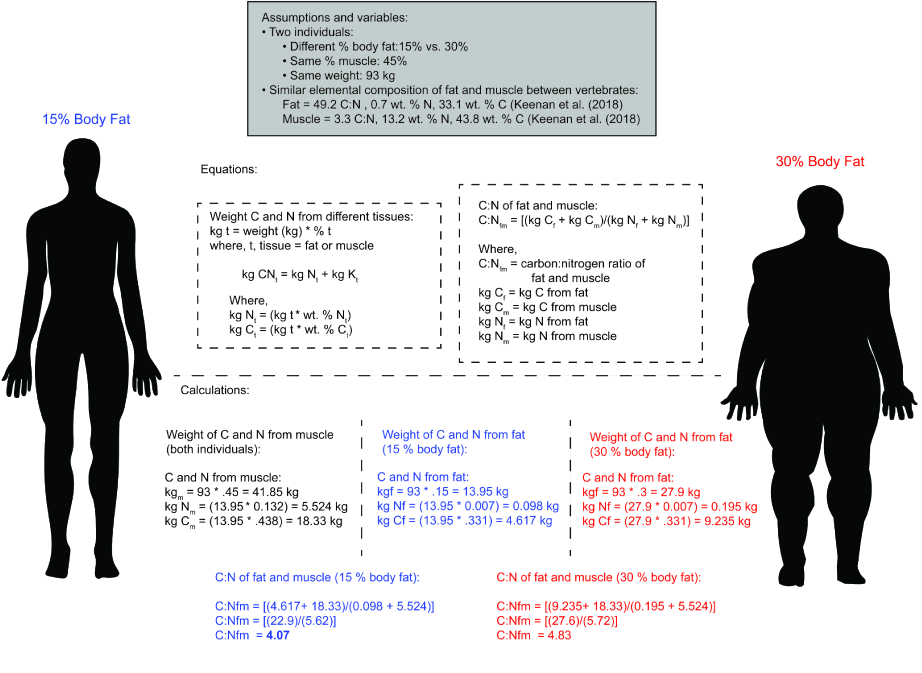

Supplement: FIG S6 [file msphere.00325-22-s0006.tif]
