# Supplementary material for: Body Mass Index (BMI) Impacts Soil Chemical and Microbial Response to Human Decomposition
Source: mSphere. 2022 Sep 22;7(5):e00325-22. doi: 10.1128/msphere.00325-22 (PMC9599287; doi:10.1128/msphere.00325-22)
Supplement: TABLE S1 [file msphere.00325-22-s0007.pdf]

		ADH	Cancer	ADH: Cancer	Cardio-vascular	ADH: Cardio	Resp-iratory	ADH : Resp	Neuro-logical	ADH : Neuro
pH LRR*	F	***12.89***	3.765	1.156	0.619	3.333	0.031	3.845	0.898	***30.79***
	p	***< 0.001***	0.068	0.284	0.442	0.069	0.862	0.051	0.357	***< 0.001***
Electrical Conductivity LRR	F	35.12	0.887	0.129	***11.65***	0.508	0.376	0.005	1.210	0.225
	p	0.237	0.348	0.811	***0.001***	0.751	0.540	0.973	0.273	0.810
Heterotrophic Respiration LRR	F	***330.1***	0.047	***8.776***	0.274	***5.077***	0.070	0.732	0.011	***6.093***
	p	***< 0.001***	0.830	***0.004***	0.606	***0.026***	0.794	0.394	0.917	***0.015***
β-glucosidase LRR	F	0.455	1.736	0.129	1.860	0.367	0.148	0.482	***6.311***	1.662
	p	0.503	0.196	0.721	0.181	0.547	0.703	0.490	***0.016***	0.203
N-acetyl-β-D-glucosaminidase LRR	F	***11.60***	0.066	0.504	2.161	0.206	0.308	0.294	0.427	0.090
	p	***0.001***	0.799	0.481	0.150	0.652	0.583	0.590	0.518	0.766
Alkaline phosphatase LRR	F	***12.26***	0.311	0.069	2.136	0.025	0.881	0.320	0.918	0.153
	p	***0.001***	0.581	0.794	0.151	0.875	0.355	0.574	0.344	0.697
leucine amino peptidase LRR	F	***16.14***	2.513	***4.201***	< 0.001	0.726	0.101	< 0.001	0.606	0.329
	p	***< 0.001***	0.122	***0.045***	0.983	0.398	0.752	0.995	0.441	0.569
16S Chao1	F	***6.914***	0.368	3.121	0.793	0.956	0.003	***4.640***	0.036	***5.237***
	p	***0.012***	0.548	0.082	0.381	0.332	0.954	***0.035***	0.851	***0.026***
16S Inverse Simpson	F	***8.172***	0.908	2.567	0.053	0.248	0.010	0.022	0.448	0.861
	p	***0.006***	0.346	0.114	0.819	0.619	0.754	0.882	0.506	0.357
ITS Chao1	F	***13.47***	1.144	0.518	0.396	0.002	< 0.001	0.583	0.017	0.274
	p	***< 0.001***	0.291	0.474	0.532	0.966	0.986	0.448	0.897	0.603
ITS Inverse Simpson	F	***6.613***	2.148	1.163	0.924	1.101	0.020	0.335	1.652	1.303
	p	***0.013***	0.285	0.285	0.340	0.298	0.889	0.565	0.204	0.258
Saccharomycetes (Relative Abundance)	F	***23.93***	1.698	0.159	0.420	0.092	1.282	1.545	0.380	0.039
	p	***< 0.001***	0.198	0.697	0.519	0.766	0.262	0.233	0.540	0.846
