# Supplementary material for: Body Mass Index (BMI) Impacts Soil Chemical and Microbial Response to Human Decomposition
Source: mSphere. 2022 Sep 22;7(5):e00325-22. doi: 10.1128/msphere.00325-22 (PMC9599287; doi:10.1128/msphere.00325-22)
Supplement: TABLE S2 [file msphere.00325-22-s0008.pdf]

			Total ADH	Season	Sex	Age	BMI (numeric)	BMI category	# of drugs (serum)	Cancer	Cardio	Resp	Neuro
Decompositoin Fluid ANOVA results	16S Chao1	F	***9.271***	1.163	3.516	0.585	2.223	0.542	0.044	0.799	0.319	0.122	1.354
		p	***0.009***	0.364	0.082	0.457	0.158	0.594	0.837	0.386	0.581	0.732	0.264
	16S Inverse Simpson	F	***6.960***	1.493	3.959	0.024	3.653	0.477	0.563	0.621	1.321	0.068	0.001
		p	***0.019***	0.266	0.066	0.878	0.077	0.631	0.469	0.444	0.270	0.798	0.974
	ITS Chao1	F	0.092	1.129	0.244	0.291	0.303	0.422	1.25	1.052	0.247	0.083	0.006
		p	0.765	0.376	0.629	0.598	0.591	0.664	0.297	0.322	0.627	0.777	0.941
	ITS Inverse Simpson	F	0.567	0.389	1.477	0.500	1.075	0.652	0.57	2.139	0.618	0.536	0.171
		p	0.464	0.763	0.244	0.491	0.317	0.537	0.466	0.166	0.445	0.48	0.685
Decomposition Fluid PERMANOVA results	16S Bray-Curtis	F	1.075	1.500	0.889	NA	NA	0.871	NA	0.722	0.395	1.812	0.898
		r^2	0.071	0.273	0.060	NA	NA	0.118	NA	0.049	0.027	0.115	0.060
		p	0.376	0.067	0.505	NA	NA	0.616	NA	0.697	0.973	0.076	0.588
	ITS Bray-Curtis	F	0.928	1.173	1.010	NA	NA	0.764	NA	1.265	0.552	0.902	0.677
		r^2	0.062	0.227	0.067	NA	NA	0.105	NA	0.083	0.038	0.060	0.046
		p	0.419	0.301	0.394	NA	NA	0.603	NA	0.229	0.794	0.431	0.528
