# Supplementary material for: Body Mass Index (BMI) Impacts Soil Chemical and Microbial Response to Human Decomposition
Source: mSphere. 2022 Sep 22;7(5):e00325-22. doi: 10.1128/msphere.00325-22 (PMC9599287; doi:10.1128/msphere.00325-22)
Supplement: TABLE S3 [file msphere.00325-22-s0009.pdf]

	Bacterial (16S)			Fungal (ITS)		
	F	N Perm	Pr(>F)	F	N Perm	Pr(>F)
Donor	3.528	999	***0.001***	0.796	999	0.711
Season	6.662	999	***0.002***	2.408	999	0.063
Sector	5.969	999	***0.002***	6.004	999	***0.002***
Treatment	22.36	999	***0.001***	1.184	999	0.270
Sex	0.168	999	0.668	0	999	0.992
Cancer	3.675	999	0.063	1.188	999	0.284
Cardiovascular	0.991	999	0.315	0.880	999	0.341
Respiratory	16.89	999	***0.001***	6.434	999	***0.009***
Neurological	4.002	999	***0.048***	1.342	999	0.212
Timepoint	7.318	999	***0.001***	11.174	999	***0.001***
BMI category	5.116	999	***0.004***	8.782	999	***0.001***
