# Supplementary material for: Body Mass Index (BMI) Impacts Soil Chemical and Microbial Response to Human Decomposition
Source: mSphere. 2022 Sep 22;7(5):e00325-22. doi: 10.1128/msphere.00325-22 (PMC9599287; doi:10.1128/msphere.00325-22)
Supplement: TABLE S4 [file msphere.00325-22-s0010.pdf]

		Pre-placement		Control		0 ADH		1500 ADH		3000 ADH		4500 ADH		6000 ADH	
		16S	ITS	16S	ITS	16S	ITS	16S	ITS	16S	ITS	16S	ITS	16S	ITS
Treatment	F	1.27	1.15												
	r^2	0.032	0.031												
	p	0.233	0.196												
Season	F			1.124	***1.271***	***1.447***	1.029	1.236	0.978	1.422	***2.297***	1.316	***2.106***	0.958	0.760
	r^2			0.184	***0.203***	***0.224***	0.171	0.209	0.173	0.169	***0.247***	0.158	***0.231***	0.324	0.275
	p			0.271	***0.033***	***0.046***	0.374	0.139	0.516	0.084	***0.001***	0.107	***0.006***	0.489	0.778
Sex	F			1.018	0.886	0.726	0.857	1.238	1.002	1.033	1.276	1.056	1.626	0.856	0.511
	r^2			0.056	0.049	0.041	0.048	0.072	0.059	0.064	0.078	0.066	0.098	0.146	0.093
	p			0.381	0.695	0.772	0.726	0.202	0.43	0.377	0.178	0.337	0.071	0.794	0.962
Age Category	F			0.983	1.051	1.158	1.064	***1.611***	***1.318***	1.19	0.887	0.875	0.934	0.877	0.698
	r^2			0.164	0.174	0.188	0.175	***0.257***	***0.220***	0.215	0.17	0.168	0.177	0.467	0.411
	p			0.483	0.34	0.211	0.312	***0.017***	***0.049***	0.187	0.685	0.722	0.560	0.725	0.782
BMI category	F			1.272	1.118	1.349	1.193	0.915	0.954	1.114	0.923	1.251	1.121	1.203	1.333
	r^2			0.202	0.183	0.212	0.193	0.164	0.17	0.205	0.176	0.224	0.206	0.375	0.4
	p			0.142	0.167	0.083	0.096	0.604	0.580	0.299	0.591	0.193	0.316	0.188	0.218
Cancer	F			0.804	0.922	0.72	0.725	0.793	0.844	0.626	0.601	1.083	0.987	0.963	1.012
	r^2			0.045	0.051	0.041	0.041	0.047	0.050	0.04	0.038	0.067	0.062	0.161	0.168
	p			0.640	0.594	0.783	0.954	0.723	0.706	0.923	0.913	0.315	0.451	0.580	0.45
Cardio	F			0.959	0.1	1.045	1.204	1.136	0.993	0.806	0.817	0.766	0.82	0.768	0.456
	r^2			0.053	0.055	0.058	0.066	0.066	0.058	0.051	0.052	0.049	0.052	0.133	0.083
	p			0.414	0.481	0.367	0.172	0.261	0.44	0.657	0.658	0.790	0.604	0.860	0.859
Resp	F			0.916	0.804	0.833	0.853	0.774	0.799	0.884	1.074	0.830	1.491	0.830	0.830
	r^2			0.511	0.045	0.047	0.048	0.046	0.048	0.056	0.067	0.052	0.090	0.052	0.142
	p			0.479	0.873	0.601	0.707	0.733	0.784	0.554	0.328	0.637	0.117	0.637	0.616
Neuro	F			1.673	1.036	0.792	0.695	0.922	0.922	0.956	0.672	0.962	0.796	1.062	2.181
	r^2			0.090	0.057	0.044	0.039	0.054	0.054	0.06	0.043	0.060	0.050	0.175	0.304
	p			0.074	0.374	0.682	0.973	0.518	0.562	0.443	0.840	0.464	0.659	0.445	0.095
